# Dietary Fibers: Effects, Underlying Mechanisms and Possible Role in Allergic Asthma Management

**DOI:** 10.3390/nu13114153

**Published:** 2021-11-19

**Authors:** Roos E. M. Verstegen, Atanaska I. Kostadinova, Zenebech Merenciana, Johan Garssen, Gert Folkerts, Rudi W. Hendriks, Linette E. M. Willemsen

**Affiliations:** 1 Division of Pharmacology, Utrecht Institute for Pharmaceutical Sciences, Faculty of Science, Utrecht University, 3584 CG Utrecht, The Netherlands; z.merenciana@students.uu.nl (Z.M.); j.garssen@uu.nl (J.G.); g.folkerts@uu.nl (G.F.); 2 Global Centre of Excellence Immunology, Danone Nutricia Research B.V., 3584 CT Utrecht, The Netherlands; Atanaska.KOSTADINOVA@danone.com; 3 Department of Pulmonary Medicine, Erasmus MC, University Medical Center, 3015 GD Rotterdam, The Netherlands; r.hendriks@erasmusmc.nl

**Keywords:** allergic asthma, gut-lung axis, microbiota, fermentable fibers, short-chain fatty acids, immunity

## Abstract

The prevalence of asthma is increasing, but the cause remains under debate. Research currently focuses on environmental and dietary factors that may impact the gut-lung axis. Dietary fibers are considered to play a crucial role in supporting diversity and activity of the microbiome, as well as immune homeostasis in the gut and lung. This review discusses the current state of knowledge on how dietary fibers and their bacterial fermentation products may affect the pathophysiology of allergic asthma. Moreover, the impact of dietary fibers on early type 2 asthma management, as shown in both pre-clinical and clinical studies, is described. Short-chain fatty acids, fiber metabolites, modulate host immunity and might reduce the risk of allergic asthma development. Underlying mechanisms include G protein-coupled receptor activation and histone deacetylase inhibition. These results are supported by studies in mice, children and adults with allergic asthma. Fibers might also exert direct effects on the immune system via yet to be elucidated mechanisms. However, the effects of specific types of fiber, dosages, duration of treatment, and combination with probiotics, need to be explored. There is an urgent need to further valorize the potential of specific dietary fibers in prevention and treatment of allergic asthma by conducting more large-scale dietary intervention trials.

## 1. Introduction

Globally, approximately 300 million people suffer from asthma and the prevalence continues to increase, making asthma the most common chronic non-communicable disease. Asthma is a heterogeneous disorder that is characterized by reversible airflow limitation, airway hyperresponsiveness (AHR), increased mucus production, and both acute and chronic airway inflammation [1,2,3]. Bronchial obstruction and hyperreactivity to allergens or various non-specific stimuli cause expiratory airflow limitation and respiratory symptoms such as wheezing, coughing, shortness of breath, and chest tightness, varying in time and intensity [2,4,5]. Different phenotypes of asthma can be identified, including allergic asthma, non-allergic asthma, late-onset asthma, obesity-related asthma, and exercise-induced asthma [6]. Type 2 allergic asthma is the most prevalent type, which develops particularly early in life, and involves allergic sensitization triggered by airborne allergens [2]. It can be challenging to provide a confident diagnosis of asthma in children under 5 years of age because episodic respiratory symptoms, including wheezing and cough, are also common in children without asthma, particularly in those that are 0–2 years old [3,6]. Nevertheless, symptoms suggestive of asthma have been listed in the Global Initiative for Asthma (GINA) guidelines [7]. These include e.g., nocturnal symptoms of awakenings, family history of atopy, and personal history of food allergy or atopic dermatitis.

Environmental and dietary factors are suggested to play a role in allergic sensitization and allergic asthma susceptibility. Important risk factors for asthma are air pollution, childhood obesity, sedentary lifestyle, and certain dietary alterations [8]. Over the years, numerous hypotheses have been set forth that link the rising incidence and prevalence of allergic asthma and other immune disorders with environmental changes, including urbanization, housing, and diet. Such changes are assumed to affect early life immune development and maturation and therefore to contribute to the sharp increase in asthma incidence [3]. An important player in immune system development and regulation is the gastrointestinal microbiota. Widespread hypotheses linking dysregulated host microbiome and immune development to increased allergy risk include the ‘hygiene hypothesis’, stating that lack of exposure to infectants lowers development of T-helper 1 (Th1) immunity [9,10], and the ‘microbiome hypothesis’ that suggests that a lack of exposure to ‘commensal, non-pathogenic microorganisms early in life’ is related to increasing asthma prevalence [11]. Following these hypotheses, the biodiversity hypothesis has been proposed [12]. This hypothesis states that urbanization due to population growth causes a loss of environmental biodiversity in terms of both macro- and microbiota. A decline in exposure to a diversity of macro- and microbiota for humans may lead to dysbiosis of the human microbiome, immune dysfunction, inflammation, and eventually to clinical disease [12].

In early life, the presence of an atopic or allergic sensitization state might increase the risk of developing inhalant-triggered hypersensitivity and/or asthma later in life, a phenomenon known as the atopic march [13]. This means that allergic asthma can be preceded or accompanied by other T-helper 2(Th2)-driven disorders from the atopic march, such as atopic dermatitis, food allergy, or allergic rhinitis [13,14]. During the first thousand days of life, both the immune system and the intestinal microbiota are being shaped. Bacterial colonization is assumed to begin in utero and its diversity established during childbirth, the first days of life, and up until two years of age, when it reaches a more stable state. Interactions between the microbiota and the host immune system during this critical window of opportunity in early life are necessary for the development, education, and maturation of the host mucosal immune system [15,16], and support regulatory T cell (Treg) differentiation [17]. Studies in germ-free mice have enabled the conclusion that microbial exposure plays a profound role in the morphological and functional development of the immune system [18]. More importantly, such studies suggest that the ability to intervene and restore immune defects that occur in the absence of microbiota is limited to a short time period in early life, underscoring the importance of the critical window of opportunity [19]. With an understanding of the importance of microbe-immune-system interactions in early-life development of the immune system and tolerance to harmless antigens, it can be hypothesized that any disruptions to early-life microbial exposure may result in potentially persistent immune abnormalities and increase susceptibility to allergic disease later in life. Consistent with this hypothesis, some studies have found an association between disturbances in early life microbiome composition and diversity and an increased risk of allergic asthma in young children [8,20], while other studies have been less clear on asthma but more clear on atopy and allergic rhinitis [21]. Susceptibility to childhood allergic asthma is, however, influenced by the neonatal gut microbiota and metabolic activity which determine the gut metabolic microenvironment which might direct T cell populations and function [3,22]. This suggests that the neonatal intestinal microbiome is an interesting target for dietary interventions which might protect against allergic airway inflammation.

Factors leading to the disruption of host microbiome composition and activity, referred to as dysbiosis, have been suggested to promote low-grade inflammation and the development of allergic disorders including allergic asthma [23]. The gut microbiota can be modified by dietary changes. For the most part, research regarding the impact of diet on the microbiota and the immune system has focused on dietary fiber because of their known effects on gut colonization, microbiota composition, and metabolic activity. The clear link between the immune system, microbiome, and fibers raises the question whether dietary fibers can play a role in allergic asthma prevention and symptom control. This review provides an overview of the current state of knowledge on how dietary fibers and their bacterial fermentation products may affect the pathophysiology of allergic asthma. We focus on how dietary fibers protect against disease development and improve symptom control of type 2 asthma, as shown in pre-clinical and clinical studies.

## 2. Pathophysiology of Allergic Asthma

Children with mild-moderate-severe allergic asthma are treated with inhaled corticosteroids and/or long-acting beta-agonists. Systemic adverse effects of corticosteroids use in children may be underestimated and can lead to failure to thrive, reduced bone density, and suppressed general well-being. Furthermore, 5–10% of asthma patients are poorly controlled or insensitive to corticosteroid treatment. Even if controlled, use of inhaled corticosteroids during infancy cannot prevent persistence of asthma later in life, particularly in sensitized infants [24]. Both human milk oligosaccharides (HMO) found in breast milk, and dietary fibers, may have the ability to affect microbiome composition and activity as well as immune development. In early life this derives from the Th2 polarized immune response towards a Th1 and regulatory T-cell driven protective immune homeostasis [25]. This may prevent the development of allergic sensitization and lower the allergic asthma risk. In cases of established type 2 asthma induced by airborne allergens, microbiome modulation by means of specific dietary fiber intervention may help to control symptom provocation and/or severity. Since specific dietary fibers may be applied to prevent and/or treat allergic asthma, it is important to recognize the different immune pathways and cells involved in the allergic sensitization and effector phases in asthma.

### 2.1. The Sensitization Phase of Allergic Asthma

The airway epithelium is the first site of contact with airborne allergens, such as pollen, pet dander, or house dust mite (HDM). Intercellular apical tight junctions form zipper-like structures that protect against aspecific leakage of macromolecules, such as allergenic proteins via the paracellular route. Therefore, beyond the protective mucus layer, the tight junction barrier is of also of great importance in providing a physical barrier. Bronchial epithelial cells (BECs) also co-ordinate mucosal immune responses in the lung. Protease activity of certain allergens, such as HDM or cockroach, can disrupt the epithelial barrier, thereby increasing its permeability [26]. Additionally, airborne allergens have the intrinsic capacity to activate BECs [27]. Upon allergen exposure, BECs can secrete cytokines, such as thymic stromal lymphopoietin (TSLP), interleukin 25 (IL-25), and IL-33, and chemokines including chemokine (C-C motif) ligand 2 (CCL2) and CCL20 that drive allergic sensitization [27,28,29]. The chemokines attract naïve dendritic cells (DCs), which are subsequently activated by BEC-derived mediators including IL-33 and TSLP [4]. Allergen-carrying DCs mature and migrate to mediastinal lymph nodes (medLN) [4,29], where they drive allergen-specific naïve T cells (Tn) to differentiate into follicular helper T cells (Tfh), which gain the capacity to produce IL-21 and IL-4 [30,31]. As a next step, these IL-4 producing Tfh cells may differentiate into allergic type Th2 cells or induce a microenvironment that supports Th2 differentiation [30,31]. Once activated, Th2 cells secrete IL-4 in the medLN resulting in further Th2 polarization and survival and instruct allergen-specific B lymphocytes to switch to immunoglobulin E (IgE) [32]. The allergen-specific Th2 cells obtain lung homing markers and by a Notch signaling-dependent mechanism they upregulate the sphingosine-1-phosphage receptor, which is essential for lymph node egress [33]. Allergen-specific Th2 cells, B cells, and plasma cells may home back to the lungs where they reside in the mucosal tissues directly underlying the BEC. IgE-producing plasma cells were shown to be present at various sites, including lymph nodes and bone marrow, but the contribution of the individual areas to IgE production remains unknown [34].

### 2.2. The Efffector Phase of Allergic Asthma

Upon allergen re-exposure in sensitized individuals, allergens that cross the epithelial lining directly cross-link IgE antibodies bound to the FcεR present especially on mast cells in the bronchial mucosa. The resulting FcεR activation subsequently induces degranulation and release of inflammatory mediators including histamine (HA), prostaglandins (PGs), and cysteinyl leukotrienes (cysLT). This release causes bronchoconstriction, mucosal oedema, and excessive mucus secretion contributing to acute asthmatic symptoms [32].

Upon re-exposure, allergen-specific Th2 cells that are further attracted to the bronchial tissue release the type 2 signature inflammatory cytokines IL-4, IL-5, and IL-13 dependent on the Th2-signature transcription factor GATA-3 [35]. These cytokines induce and potentiate eosinophilic airway inflammation, goblet cell hyperplasia, and/or excessive mucus secretion, and proliferation of IgE-producing plasma cells resulting in the development of chronic symptoms due to epithelial barrier disruption, airway remodelling, and airway hyperreactivity [3,32,35,36]. As well as T cells, another lymphocyte population, the group 2 innate lymphoid cells (ILC2s) have the capacity to produce Th2 cytokines, particularly IL-5 and IL-13. Unlike lymphocytes of the adaptive immune system, ILC2s do not express antigen-specific receptors, but instead can be activated by a wide range of molecules, including the pro-inflammatory cytokines IL-33 and TSLP produced by BECs upon allergen exposure [37]. ILC2s can be activated prior to the induction of T cells and thereby act as an important early innate source of type 2 cytokines. This has been shown in experimental asthma models using allergens such as papain or the fungal allergen Alternaria which act as pro-inflammatory triggers at the mucosal tissue of the respiratory tract [38]. Nevertheless, HDM-driven allergic airway inflammation critically depends on Th2 cells, and cytokine production by ILC2 mainly sets off after the peak of T cell activation [38]. Interestingly, Th2 cells produce IL-13 as well upon exposure to epithelial cytokine IL-33, independent of T cell receptor stimulation [39].

Apart from Th2, other Th subsets are associated with asthma pathogenesis, albeit these, in general, play a less prominent role in type 2 allergic asthma. Th17 cells produce IL-17, which contributes to airway hyperreactivity and the accumulation of neutrophils in the lung, and produces granulocyte macrophage colony stimulating factor (GM-CSF), which may drive airway inflammation [40,41]. Furthermore, as the main source of IL-9, Th9 cells promote activation of eosinophils and mast cells and stimulate epithelial mucus production [42].

As well as the role of T effector cells in driving eosinophilic airway inflammation, a dysfunction in Tregs has been implicated in allergic asthma. Tregs play a key role in controlling aberrant immune activation and contribute to immune homeostasis. There are different subsets of Tregs, including Th3 cells, T_R_1, and natural and induced *FOXP3*-expressing CD4^+^CD25^+^ Treg, all of which secrete immune-suppressive cytokines, such as IL-10 (T_R_1 and CD4^+^CD25^+^) and TGF-b (Th3 and CD4^+^CD25^+^) [43]. One review article highlighted that, although both increased and decreased numbers of Tregs are found in asthma patients, it is well documented that asthma patients have less functional Tregs in comparison to healthy controls [44]. This includes declined induction of allergen-specific Tregs, impaired suppressive function of Tregs, or a combination of these two.

## 3. The Gut-Lung Axis

One key factor that can influence host mucosal immunity is the microbiota and its contribution to health and disease is increasingly recognized. These microbes have important functions, including the fermentation of indigestible dietary components such as dietary fiber to generate unique metabolites, nutrients, and vitamins that can impact immune reactivity. In addition, they maintain immune homeostasis by supporting Treg development and inflammatory responses against pathogens [45]. Microbiota abundance and diversity increases along the gastrointestinal (GI) tract, but the most metabolically active, fiber-fermenting populations, are harbored by the colon [46]. The microbiome community composition in the small intestine shifts more rapidly than the colon community and can ferment simple rather than complex carbohydrates due to the rapid transit time of food [47]. The microbial composition in the GI tract can differ between people due to genetics or environmental, lifestyle, and dietary factors, as well as at different life stages [48]. For instance, in early life, neonate microbiota is of low diversity and richness with relative higher abundance of the Proteobacteria and Actinobacteria phyla. In vaginally delivered and breastfed new-borns, the *Bifidobacterium* genus predominates and persists until solid food introduction and weaning [49]. The *Lactobacillus* genus is also found in the infant gut, and both viable bifidobacteria and lactobacilli have been detected in breastmilk [50]. This points to the important contribution of human milk to the initial establishment of the microbiota in newborns and provides a foundation for balanced infant gut colonization. In the period between two and five years of age, the gut microbiota evolves and stabilizes to resemble the more diverse composition observed in adults with predominating Firmicutes and Bacteroidetes phyla [45,48]. Changes in the composition or diversity of the microbiota may have a serious impact on the host [48] by increasing the risk of disease.

Healthy lungs were long thought to be sterile, but it is currently recognized that even neonates have a lung microbiome. As with the intestinal microbiota, the load and diversity of the microbiota in the upper respiratory tract differs from the lower respiratory tract [51]. Similarities between the bacterial communities of the oral cavity and the lungs indicate that the microbiota of the latter is influenced by the microbiome of the oral cavity and GI-tract due to micro-aspiration, the unintentional aspiration of very small amounts of gastric contents [51,52]. The most prevalent communities present in the lungs are *Streptococcus*, *Prevotella,* and *Veillonella.* Similar to the GI-tract, the phyla Bacteroidetes and Firmicutes are part of the ‘core’ microbiota in the lung [45]. Studies in early life have indicated the rapid colonization of the lung and have suggested that the airway microbiome reaches its mature diversity at 2–3 months after birth [53]. Early changes in the lung microbial composition determine the local immunological environment and can increase susceptibility to allergic airway inflammation, while in chronic disease it could lead to exacerbation and decline of lung function [54]. This indicates that early life is an important window of opportunity for intervention and reprogramming.

There is now an increasing recognition of crosstalk between the lungs and the gut, known as the ‘the gut-lung axis’. Cross-regulation of gut-lung immunity is evident and it is understood that the intestinal microbiota might directly modulate the immune responses to invading pathogens in the lung [55,56]. Established inflammation in the gut can predispose mice to both allergen-specific and non-specific airway responses [57]. In line with this, oral treatment with bacterial extracts was shown to suppress airway inflammation in mice by targeting DCs and ILC2s in the lung, most likely via Toll-like receptor (TLR) signaling, as well as by increasing Treg numbers in the trachea [58,59]. These Tregs were originally induced in the intestines and relocated via the bloodstream to the airways [58]. Studies in humans with oral and sublingual allergen-specific immunotherapy for allergic disease have reported that locally and peripherally generated Tregs migrate to distant effective sites, such as the lungs and nose, and lead to reduced Th2-driven effector responses in allergic individuals [60]. Modulation from the lungs to the gut is possible as well. DCs in the lungs were found to upregulate the gut-homing integrin α4β7 and the chemokine receptor 9 (CCR9) expression on T cells, leading to T cells migrating towards the intestine to protect against pathogenic infection [61]. Interestingly, CCR9 was upregulated in Treg cells that migrated from the gut to the trachea [58]. Another possible mechanism by which crosstalk and systemic tolerance can be induced is through antigens that reach distant lymph nodes via the bloodstream [62]. Associated with the concept of the ‘common mucosal immune system’, crosstalk between the gut and lungs could also be hypothesized to take place through cross-colonization of the microbiota. Gut bacteria might travel to the lungs by micro-aspiration or through gastroesophageal reflux [63]. Conversely, lung microbiota could be deposited in the gut upon swallowing mucus that is propelled out of the lungs [64]. As healthy lung microbiota are vital for respiratory health, both the gut and lung microbiome are an attractive target for future preventive and therapeutic strategies for fighting gastrointestinal as well as respiratory diseases, including allergic asthma.

## 4. Fiber Types, Functions and Metabolites

**Figure 1:**
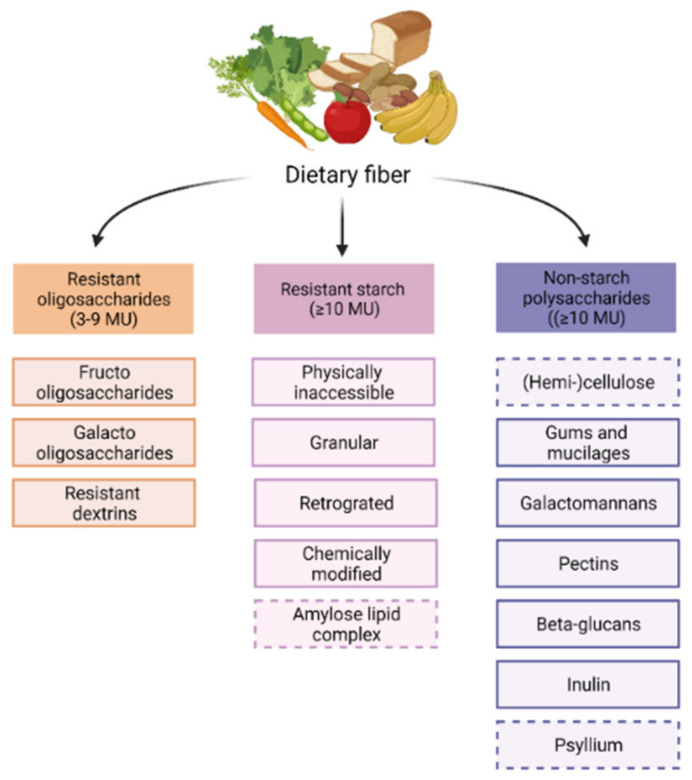
Chemically based dietary fiber classification. Dietary fibers are found in fruits, vegetables, whole grains, legumes, and nuts and seeds. There are three groups in which fibers are chemically classified: Resistant oligosaccharides have 3–9 monomeric units. Resistant starches, and non-starch polysaccharides, have 10 or more monomeric units. Dashed boxes are non-fermentable fiber.

Dietary fibers have the capacity to modulate the gut microbiome. Although all dietary fibers share the feature of being non-digestible in the small intestine, they are still quite heterogenous. Dietary fibers are classified based on their origin or physiochemical properties, for instance, solubility, viscosity, and fermentability [65]. The chemically based division classifies dietary fibers into resistant oligosaccharides (ROs), including fructo-oligosaccharides (FOS) and galacto-oligosaccharides (GOS), resistant starch (RS) and non-starch polysaccharides (NSPs) [66], as shown in Figure 1.

As all fibers have different properties, they also exert different functional effects in the GI-tract contributing to metabolic alterations such as modifying cholesterol levels and improving glycemic control [65,66]. Insoluble fibers, such as cellulose and hemicellulose, are not fermented but act as bulking fibers, instigating faster transit times and improved regularity of stool [66]. In this review, the primary focus will be on fibers that are soluble and fermentable, because the effects of their metabolites on immune function have been described in most detail. This includes all ROs, all RS, except for amylose-lipid complexes, and the NSPs, except for (hemi-)cellulose and psyllium.

Gut bacteria express enzymes that can break down specific glycosidic bonds in the structure of undigestible fibers. Bacteria vary in the type and number of enzymes they produce, the fibers they ferment, and consequently the metabolites that are produced. Therefore, a diet rich in various fibers supports a diverse gut microbiome in humans [65]. Bacteria profit from fiber fermentation by obtaining energy. In the absence of fibers, bacteria would use the glycoprotein-rich mucus layer of the gut as an alternative energy source, thereby disrupting this protective barrier [67]. Humans can benefit from the fermentation (end-)products or metabolites, of which the short-chain fatty acids (SCFA) acetate, propionate, and butyrate are the most abundant [65]. Acetate, propionate, and butyrate are typically found in the gut, and specifically are abundant in the cecum and proximal colon, in a ratio of 80:10:5 in children [68] and 60:20:20 in adults [69]. The SCFAs are either used by the microbiota or absorbed via passive diffusion or various sodium-coupled monocarboxylate transporters (SMCT) and monocarboxylate transporters (MCT) [70]. Following absorption, SCFAs are utilized by intestinal epithelial cells (IECs) to produce ATP (mainly butyrate) or are systemically distributed via the blood [69,71]. Total SCFA concentrations in the blood vary from ±375 µmol/L in the portal blood to ±79 µmol/L in the peripheral blood. Acetate is the most prominent SCFA in the blood and can cross the blood-brain-barrier [69,72]. The SCFA concentration in the lungs has not been well elucidated yet. Based on the few studies that have investigated this matter, it can be anticipated to be somewhere between 1–10,000 µmol/L, with acetate predominantly present [73,74,75]. SCFAs can bind to specific receptors and act as signaling molecules throughout the whole body via different mechanisms.

## 5. Mechanisms of Action of SCFAs

**Figure 2:**
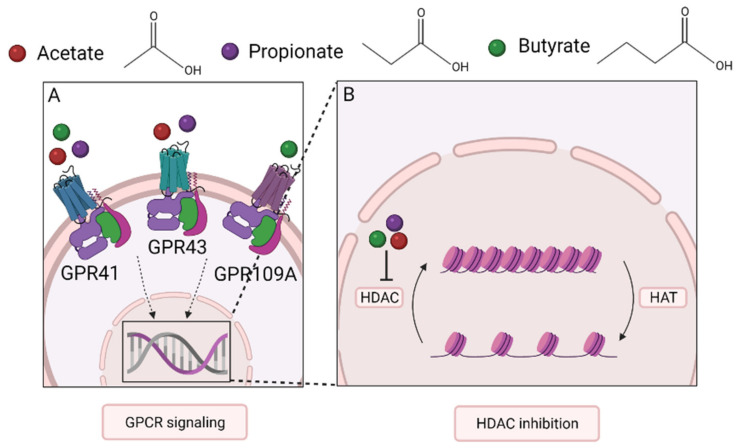
Mechanisms of action of SCFA. At present two mechanisms are recognized by which SCFA influence immune function. (**A**) SCFAs bind to transmembrane GPCRs on epithelial and immune cells to induce downstream signaling. GPR41 is bound by all three SCFAs, GPR43 mainly by acetate and propionate, and GPR109A mainly by butyrate. (**B**) SCFAs are also known to inhibit the enzyme HDAC. HDAC deacetylates histones which suppresses gene expression. HAT has the opposite effect. SCFA, short-chain fatty acid; GPCR, G-protein coupled receptor; HDAC, histone deacetylase; HAT, histone acetyltransferase.

SCFAs act as a bridge between the microbiota and the immune system and maintain immune homeostasis. Evidence has accumulated that SCFAs affect many cell types, including dendritic cells, T cells, ILC2s, mast cells, and eosinophils, which may also apply to the lungs. Even though the mechanisms involved are incompletely understood, G-protein-coupled receptor (GPCR) activation and histone deacetylase (HDAC) inhibition appear to play a major role in their mechanism of action [71] (Figure 2).

### 5.1. GPCRs and Downstream Signaling Pathways

GPCRs are transmembrane receptors found on cells throughout the whole body. The GPCRs associated with SCFAs are GPR43 (also called free fatty acid receptor 2 (FFAR2)), GPR41 (FFAR3), and GPR109A [45,71]. Downstream pathways involve both pro- and anti-inflammatory signaling cascades [45]. Alterations in the activation of these pathways have been linked to changes in the metabolic system and the onset of metabolic diseases [76]. GPR43 is mainly bound by acetate and propionate and found in various cell types, including cells of the intestinal epithelial lining and cells of the associated mucosal immune system, including DCs, Tregs, mast cells, eosinophils, and neutrophils [71,76,77]. All three SCFAs bind to GPR41, which shows a largely similar expression pattern to GPR43, but is also present in cells in the bone marrow and lymph nodes [71,76,78]. Stimulation of either of these two receptors can initiate activation of the mitogen-activated protein kinase (MAPK) pathway [45].

GPR109A, also known as niacin receptor 1 (NIACR1), is activated by butyrate [71]. In contrast to GPR43 and GPR41, which are assumed to be only activated by SCFAs, GPR109A has vitamin niacin (vitamin B3) as its ligand [79]. This receptor is present, amongst others, on the apical site of the colon epithelium, DCs, macrophages, monocytes, and neutrophils, but not on lymphocytes [71].

In addition, the intermediate-fermentation products, lactate and succinate, bind to some GPCRs, such as GPR81 or GPR91. However, due to its conversion into propionate or butyrate lactate it is not expected to function as a GPCR-ligand outside the gut. There is a lack of evidence that succinate functions as a signaling molecule [71]. Therefore, these metabolites will not be discussed in further detail in this review.

### 5.2. HDAC Inhibition and Epigenetic Imprinting

The second way via which SCFAs influence immune function is by acting as inhibitors of HDAC, which modify the epigenome through chromatin remodeling [80]. The epigenome entails heritable molecular modifications to the DNA and histones (octameric proteins that organize chromatin into nucleosomes), which can be passed on from cell to cell, as they template their own replication, and control gene expression [81,82]. It defines cell identity and function without alterations in the DNA sequence, thereby imposing a cellular differentiation program or reflecting an adaptation to external environmental factors [83]. Histone modification by acetylation or methylation is one of the epigenetic mechanisms responsible for modifying gene expression, alongside DNA methylation. HDACs hydrolyze acetyl groups from lysine residues of histone proteins and thereby support the formation of condensed chromatin. They antagonize the activity of histone acetyltransferases (HATs) that enhance chromatin accessibility and facilitate transcription. Enhancers and promotors of active genes generally harbor the active histone 3 acetylation marks at K9 and K27 (H3K9ac and H3K27ac) [84]. Modulation of histone acetylation and deacetylation through environmental factors, including dietary components, may possibly prevent disease and maintain health by changing the accessibility of genes to the transcription machinery.

The epigenome provides genome-wide information on developmental history and prior stimulation, presents identity and function, and future potential and cellular plasticity in health and disease. Most of this information cannot be obtained by gene expression or genome-wide association studies (GWAS). Numerous asthma-associated gene loci have been identified by GWAS, but these loci provide limited insight into the underlying mechanisms and cell types affected [85,86]. However, epigenetic studies have identified (i) regions of differential DNA methylation associated with allergic inflammation [87,88], (ii) epigenetic differences in Th2 cells between asthma patients and healthy individuals, and (iii) an enrichment of asthma-associated single-nucleotide polymorphisms in gene regulatory elements of Th2 cells [89,90]. Moreover, epigenome analysis has shown strong correlations between gene regulatory mechanisms in ILC2s and the genetic basis of allergic asthma [91]. Recently, the effects of SCFA on epigenetic imprinting in ILC2s and mast cells have been revealed, which will be further discussed in the section below, describing the effects of SCFAs on allergic-asthma-related immune outcomes in detail.

## 6. Immune Effects SCFAs

**Figure 3:**
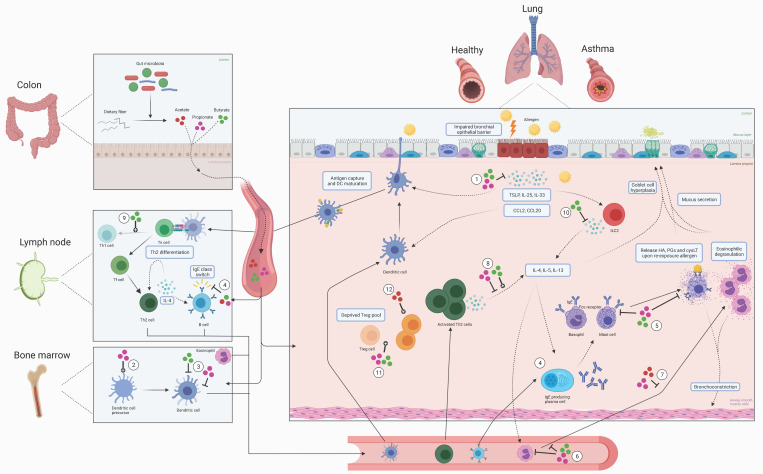
Effects of SCFAs on the immune response in allergic asthma. Upon allergen exposure the airway epithelial barrier is damaged and activated, leading to type 2 driving mediator release and DC activation. These DC capture allergens and migrate to the mediastinal lymph nodes, where allergen presentation by DCs stimulates Th2 differentiation. Th2 cells instruct allergen-specific B cells to produce IgE (isotype switching). Th2 and plasma cells migrate to the mucosal tissue in the bronchi. Activated Th2, ILC2 and allergen-IgE stimulated mast cells induce eosinophil infiltration (eosinophilic airway inflammation) and asthma symptoms. Tregs in asthma patients are dysfunctional. (**1**) Propionate and butyrate increase tight junction protein expression and inhibit MAPK pathways, thereby restoring/inhibiting epithelial barrier dysfunction. (**2**) Propionate increases DC and macrophage precursor numbers (**3**) Propionate reduces DC activation and lowers MCHII expression, butyrate hampers DC maturation, lowers its migration efficacy and antigen capture efficiency. (**4**) Acetate, propionate, and butyrate reduce IgE isotype switching. (**5**) Propionate and butyrate hamper expression of FcεR-related genes, thereby reducing allergen/IgE triggered degranulation and asthma symptoms. (**6**) Propionate and/or butyrate may induce apoptosis in eosinophils, hinder migration from the bone marrow, and prevent adhesion of eosinophils to endothelial cells, hampering their infiltration into the lungs. (**7**) Acetate and propionate can also prevent airway infiltration by eosinophils. (**8**) Propionate decreases IL-13 release by Th2 cells, which may reduce goblet cell hyperplasia, mucus production, chronic inflammation, and allergen specific-IgE levels, while butyrate promotes cytokine production. (**9**) Butyrate induces expression of Th1 transcription factor T-bet and IFNγ. (**10**) Butyrate inhibits ILC2-mediated cytokine release. (**11**) Propionate and butyrate facilitate generation of extrathymic Tregs, and butyrate promotes Treg differentiation. (**12**) Acetate increases acetylation FOXP3 promotor. Full arrows with fading represent transition. Full arrows without fading represent migration. Dotted arrows with fading represent cytokine or chemokine induced stimulations. TSLP, thymic stromal lymphopoietin; IL, interleukin; CCL, chemokine ligand; Th2, T helper cell type 2; Tf, follicular T cell; IgE, immunoglobulin E; ILC2, type 2 innate lymphoid cells; HA, histamine; PGs, prostaglandins; cysLT, cysteinyl leukotrienes; Fcε, fragment crystallizable region epsilon; Treg, regulatory T cell. The possible mechanisms of action shown in this cartoon are a compilation of SCFA effects derived from in vitro and/or in vivo studies.

To date, many effects of SCFAs on immune cells and their mediators in the context of allergic asthma have been identified. An overview is presented in Figure 3.

### 6.1. Sensitization Phase of Allergic Asthma

#### 6.1.1. (Airway) Epithelium

The epithelial layer, either in the gut or lung, is involved in maintaining health. It provides a physical barrier, comprising the mucus layer and tight junctions between epithelial cells, against intruders and modifies mucosal immune function via secretion of specific cytokines and chemokines when activated by pathogens. Failure of function or unwanted activation of the epithelial barrier at mucosal sites of the body has been linked to immune-related disorders. Most importantly, a leaky gut epithelium can contribute to inflammation, even at distant locations [92]. Butyrate profoundly promotes the intestinal epithelial barrier function and all three SCFAs modify mucosal immunity [93]. The airway epithelium is the first line of defense against airborne allergens such as HDM that can cause allergic airway disease (AAD). In a human bronchial epithelial cell (HBEC) cell line 16HBEC, the airway epithelial barrier dysfunction, caused by exposure to HDM and cytokines including IL-4 and IL-13, could be completely restored by propionate and butyrate, as these SCFAs increased the expression of tight junction proteins and inhibited certain MAPK pathways [94]. HDAC inhibition in HBECs from asthmatic patients restored the epithelial barrier function, as determined by transepithelial electric resistance and tight junction level measurements of HBECs to the level of control subjects [95]. Regarding allergic rhinitis patients, HDAC inhibitors did not only restore epithelial function in vitro, but also averted allergic airway inflammation by supporting nasal tight junction expression when applied endonasally in HDM-sensitized mice [96]. This latter might be due to the HDAC inhibitory potential of SCFAs.

#### 6.1.2. Dendritic Cells

DCs are key players in allergic sensitization, mainly due to their role in antigen-presentation and T-cell differentiation [4]. Butyrate can hamper DC maturation, rendering a tolerogenic phenotype with reduced migration efficacy and capacity to activate T cell proliferation [97,98,99]. Despite impeding DC maturation, DCs increased the production of the Th17-stimulating cytokine IL-23 in vitro upon exposure to butyrate [99]. Intraperitoneal injection with propionate in mice during HDM sensitization and challenge, increases common DC and macrophage (MDPs) in the bone marrow [78]. Propionate did not influence proportions of the individual DC subpopulations in lung-draining lymph nodes but reduced the activation state of CD11b^hi^ DCs [78]. Additionally, the fraction of newly recruited DCs in the lung upon HDM challenge was reduced and these DC showed a diminished expression of MHCII and the activation marker CD40, compared with mice injected with saline. As a result, these DCs were less effective in reactivation of Th2 effector cell proliferation, protecting the mice against HDM-induced allergic airway inflammation [78]. These findings imply that SCFAs could diminish DC activity in the lung, via modifications of DC progenitor cells in the bone marrow, thereby downsizing their inflammatory potential.

#### 6.1.3. B Cells and Immunoglobulins

Allergen-specific antibodies of the IgE subclass, secreted by plasma cells, have the capacity to opsonize mast cells. IgE is required for allergen-induced elicitation of allergic symptoms as a result of mast cell activation. Supplying a mixture of acetate, propionate, and butyrate in the drinking water of vancomycin-treated mice with ovalbumin(OVA)-induced allergic asthma revealed that an SCFA mixture abrogated the rise in serum IgE, thereby preventing sensitization. The proposed underlying mechanism of this effect pointed towards reduced germline transcription over the immunoglobulin heavy chain ε switch region, which is associated with class switch recombination to IgE, predominantly in B cells in the medLNs [98]. Interestingly, although not being the most common SCFA, pentanoate (C5) enhanced the activity of the mechanistic target of rapamycine (mTOR) kinase in regulatory B cells, leading to increased IL-10 secretion and reduced cell death. This effect has been linked to the HDAC inhibitory effects of pentanoate [100].

### 6.2. Effector Phase of Allergic Asthma

#### 6.2.1. Mast Cells

Mast cells are key effector cells that can initiate and propagate inflammation in allergic asthma [101]. Specific binding of allergens to FcεR-bound IgE triggers degranulation, resulting in the rapid release of HA, eicosanoids, and an array of proteases and cytokines. An ex vivo experiment by Folkerts et al. demonstrated the potency of propionate and butyrate to impair IgE-mediated mast cell degranulation. The underlying mechanism reported involved alteration of histone acetylation resulting in hampered expression of genes crucial for FcεRI-mediated degranulation [102]. When precision-cut lung slices from the lower airways of OVA-sensitized guinea pigs were exposed to OVA, treatment with butyrate attenuated IgE/allergen-induced airway smooth muscle contraction in association with abolished histamine release by mast cells [102]. Propionate and butyrate, but not acetate, reduced degranulation, as well as IL-6 production, triggered by IgE, compound 48/80, or substance P, in primary mouse bone-marrow-derived mast cells and in human peripheral blood mononuclear cell-derived, ex vivo differentiated, mast cells [102]. All these inhibitory effects of SCFA were independent of signaling through the GPR41/43 or intracellular peroxisome proliferator activated receptor γ (PPARγ), but instead were associated with HDAC inhibition. Expression profiling of human mast cells revealed that >700 genes were upregulated and >900 genes were downregulated following in vitro butyrate treatment [102]. Butyrate exposure significantly reduced transcription of several genes that play a key role in FcεR signaling, such as Bruton’s tyrosine kinase (*BTK*), spleen tyrosine kinase (*SYK*), and linker of activated T cells (*LAT*). Downregulation of these genes also occurred upon butyrate exposure to IgE/antigen-stimulated mast cells. Akin to previous studies [103], butyrate was shown to induce elevated global histone acetylation but decreased acetylation at the transcription start sites of *BTK*, *SYK*, and *LAT*, as well as other key genes involved in mast cell activation [102]. Parallel findings were reported by Krajewsky et al., who showed that the HDAC inhibitor, trichostatin A (TSA), decreased FcεR expression, degranulation capacity, and survival of murine mast cells [104]. These findings may appear to conflict with the general notion that HDAC inhibitors act by reactivation of epigenetically silenced genes, particularly in the context of their anti-tumorigenic properties [105]; however, butyrate has the capacity to decrease histone acetylation around transcript start sites and to downregulate gene expression, in line with downregulation of the expression of key oncogenes [103].

#### 6.2.2. Eosinophils

An interesting in vitro study by Theiler et al. demonstrated apoptosis-inducing effects of propionate and butyrate in human eosinophils of allergic donors via the HDAC class IIa-selective inhibitor MC1568. Furthermore, propionate and butyrate could prevent adhesion of eosinophils to endothelial cells and butyrate could hinder eosinophil migration. Murine experiments confirmed that this leads to less eosinophilic airway inflammation and improved AHR [77]. In vitro, butyrate inhibits ILC2 proliferation and cytokine production through its HDAC inhibitory actions, thereby indirectly preventing eosinophilic inflammation [106]. A murine study investigating acetate and propionate supplementation in drinking water revealed the potential of these SCFAs to prevent HDM-induced airway infiltration by eosinophils, thereby protecting the mice against allergic airway inflammation. Propionate exerted its actions in a GPR41-dependent manner [78]. These findings are in line with preclinical and clinical studies showing reduction of pulmonary eosinophil infiltration upon interventions with dietary fiber to be discussed in the next section of this review.

#### 6.2.3. T Cells and ILC2s

SCFAs affect T cells in many ways. Acetate and propionate were found to induce genes indirectly related to T cell differentiation via HDAC inhibition, and, via this route, to support Th1 and Th17 development [107]. On the other hand, butyrate, and less potently propionate, diverted the fate of naive CD4+T cells from Th9 cells into *FOXP3*+ Tregs under in vitro Th9-skewing polarization conditions. In vivo, butyrate reduced the number of IL-9-expressing T cells in the lungs [108]. Although these results are noteworthy, other groups of T cells may be of greater importance in relation to type 2 allergic asthma.

#### Th2 Cells

In most patients, allergic asthma presents as an eosinophilic airway inflammation that is mediated by Th2 cells, which induce IgE class switching (IL-4), recruit eosinophils (IL-5), and provoke smooth muscle hyperreactivity, goblet cell hyperplasia, and mucus production (IL-13) [3]. Butyrate and propionate had opposite effects when naïve CD4+ T cells were differentiated in vitro under Th2-polarizing conditions in the presence of IL-4 and anti-IFNγ antibodies [108]. Whereas butyrate induced a small but significant increase in the frequency of IL-13-expressing T cells, propionate induced a decrease. No significant differences were observed in the proportions of IL-4 or IL-5-expressing T cells in these cultures. In another study, butyrate enhanced Th2 polarization both in human and murine in vitro cultures and promoted Th2 cytokine production in an aspergillus allergen-challenged murine asthma model [109]. Remarkably, the expression of GPR41 increased upon IL-4 stimulation of Jurkat T cells, indicating that SCFA may act directly on Th cells to promote Th2 polarization, involving a positive feedback loop. By contrast, in a murine model of HDM-driven allergic airway inflammation, mice that were prophylactically treated with propionate (intraperitoneal) displayed reduced inflammatory cell lung infiltration, IL-13 mRNA expression, goblet cell hyperplasia, mucus production, and serum total IgE levels [78], demonstrating the capacity of SCFAs to reduce Th2 cell responses. Experiments in GPR41-deficient mice established that this protective effect by propionate depended on GPR41 signaling. Butyrate was capable of inducing expression of the key Th1 transcription factor T-bet, which depended on its HDAC inhibitory activity. Even under Th2-promoting conditions, butyrate triggered induction of IFNγ and T-bet, and consequently lowered expression of IL-4 and GATA-3, the key transcription factors of Th2 cells [110]. As mentioned above, propionate and butyrate attenuate DC activation and migratory capacity, thereby profoundly hampering T cell activation and Th2 effector cell development [78,98]. Indirectly, in vivo these effects on DCs might be inherent to the reduced Th2 polarization that follows from prophylactic SCFA treatment.

#### ILC2s

ILC2s are a rare population of lymphocytes that, analogous to Th2 cells, are major producers of type 2 cytokines and express high levels of the signature transcription factor GATA-3 [35,111]. Although many aspects of human ILC2 biology are still unclear, recent observations strongly support a role for ILC2 in several diseases of the respiratory system including asthma [37].

Butyrate, but not acetate or propionate exposure to cultured murine lung ILC2 inhibited cellular proliferation and transcription of GATA-3, IL-5, IL-9, and IL-13 [106,112]. Systemic or intranasal administration of butyrate following *Alternaria alternata* exposure ameliorated ILC2-mediated airway inflammation and hyperreactivity in mice, marked by reduced GATA-3, IL-5, and IL-13 expression. Similarly, increased in vivo SCFA levels through a high-pectin diet modulated the response to IL-33 in the lung, resulting in reduced expression of GATA-3, IL-5, and IL-13 in ILC2s. Butyrate also reduced type 2 cytokine production in human ILC2s [106,112]. Butyrate augmented H3 acetylation levels, implying that it acts as an HDAC inhibitor in ILC2s. This is consistent with previous observations that the HDAC inhibitor trichostatin A downregulated the number of ILC2 expressing IL-5 and IL-13 following *Alternaria*-extract challenge [113]. In contrast, GPR41/GPR43 activation did not affect pulmonary ILC2 function [106] and GPR43 was even found to support ILC2 expansion in the colon in response to cytokine stimulation [114]. Finally, evidence has been provided that SCFAs can modulate oxidative phosphorylation and glycolytic metabolic pathways in pulmonary ILC2s [112]. Taken together, it has been convincingly shown that butyrate dampens ILC2 activity, at least partly by decreasing GATA3 transcription, which was not the case in Th2 cells. These findings indicate that the control of GATA3 gene expression in ILC2 and Th2 cells involves different regulatory elements. Very recently a GATA3 enhancer was identified that is necessary for ILC2 development and function, but largely dispensable for Th2 cell differentiation. It is attractive to speculate that the activity of this enhancer would contribute to the opposing direct effects of butyrate on ILC2 and Th2 cells [115]. Regulatory elements that are directly affected by butyrate-driven changes in histone acetylation in the GATA3 locus, as well as for other key genes expressed in ILC2s, remain to be identified.

#### Tregs

The control of potentially harmful Th2 cells and ILC2s is thought to require active immunosuppression by Tregs expressing the signature transcription factor *FOXP3*. To date, various mechanisms by which gut microbial products regulate inflammatory responses and Treg function have been identified. However, the effects of SCFAs on Tregs in the lung are somewhat ambiguous. Both butyrate and propionate, but not acetate, facilitate the generation of extrathymic induced Tregs (iTregs), depending on histone H3 acetylation at the *FOXP3* intronic enhancer conserved non-coding sequence-1 (CNS1) [17,116]. In another study, acetate-enriched drinking water was found to increase acetylation at the *FOXP3* promotor, almost certainly through HDAC9 inhibition, as HDAC9-deficient mice were highly resistant to the development of HDM-mediated allergic airway inflammation [117]. In a murine study, butyrate showed decrease in the expression of pro-inflammatory cytokines by DCs and enhanced their ability to promote Treg differentiation via inhibition of transcription factor RelB [17]. Nevertheless, in contrast to butyrate, in certain in vitro [116] and in vivo [17] experiments, the SCFA acetate did not influence Tregs. Trompette et al. also could not associate the protective effects of propionate in murine allergic lung inflammation with Treg numbers [78]. Several studies did, however, find SCFAs to enhance the size of the colonic Treg pool that could protect against colitis in mice in a GPR43-dependent manner [116,118,119]. As discussed earlier, immune cells from the gut can exert functions elsewhere in the body, such as the lung. Taken together, there is evidence that SCFAs support the generation of extrathymic iTregs through effects on DCs. In this context, SCFA supplementation may improve Treg-mediated immunomodulation not only in the intestine but also in peripheral tissues such as the lung [17].

#### 6.2.4. Macrophages and Neutrophils

Macrophages and neutrophils are not prominently involved in the pathophysiology of allergic asthma but have impact in chronic and severe uncontrolled type 2 asthma. SCFAs also affect these cells. While acetate increased the production of reactive oxygen species (ROS), including superoxide and hydrogen peroxide, in rat, but not in human, neutrophils in vitro, butyrate diminished ROS production in both rat and human neutrophils [120]. Acetate increased cytoplasmic calcium mobilization and protein kinase C (PKC) activity which may support ROS generation, while butyrate possibly inhibited ROS formation via GPR43 activation. SCFAs increased the expression of GPR43 and GPR41 on pulmonary neutrophils and macrophages, accompanied by reduced inflammatory IL-8 production [121]. Regarding macrophages, one in vitro study showed butyrate to alter macrophage differentiation since it inhibited upregulation of CD16, CD86, and MHCII, thereby decreasing their antigen-presenting efficacy [97]. Additionally, butyrate decreased the production of pro-inflammatory nitric oxide, IL-6, and IL12p40 by bone marrow-derived macrophages (BMDM). Butyrate exerted its effects via H3K9 acetylation at the promotor regions of the genes encoding the cytokines, and not via GPR43 or GPR109A that are present on BMDMs. These results were confirmed in mice [122].

## 7. Preclinical Studies on the Effects of Dietary Fibers on Asthma

Multiple murine studies have addressed the protective effect of dietary fiber in allergic asthma. These studies used different allergens and focused on asthma prevention and asthma symptom relief mainly in acute, but also in chronic asthma models. Furthermore, different types and dosages of fibers were investigated. Most preclinical studies focused on the effects of the ROs, GOS and FOS, or the NSP pectin. Interestingly, to the best of our knowledge the effects of inulin in rodent asthma models have not been reported, while this NSP is already used in clinical studies. Inulin has a wide range of fiber sizes (degree of polymerization (DP) 3–60). FOS (oligofructose) can be derived from inulin but is more size restricted and can be divided into short chain FOS (DP2-8, oligofructose) and long chain (lc)FOS (DP > 23) which may differentially modify microbiome composition and/or activity. It needs to be stressed that fibers with a lower DP can be hydrolyzed and fermented more quickly than fibers with a higher DP [123].

Murine allergic asthma models most frequently make use of OVA allergen, derived from chicken egg, or HDM, an airborne allergen, and often the cause of allergic asthma development in childhood. Although OVA is a model allergen often used to induce allergic airway inflammation in rodents, it is not of clinical relevance for human asthma [124]. Additionally, even though OVA challenge occurs via the nose, OVA sensitization is applied via the intraperitoneal route and requires adjuvants, such as aluminium hydroxide (alum), to induce allergic inflammation. These adjuvants could possibly interact with therapeutic substances [124,125]. Despite the existence of adjuvant-free protocols for OVA-driven asthma models [125], most studies discussed in this review made use of an adjuvant. The allergen HDM is of greater clinical relevance for allergic asthma. Moreover, this allergen has proven to sensitize and induce allergic inflammation when inhaled via the nose, and does not require an adjuvant [124]. Beyond allergens, intranasal IL-33 administration has also been applied, because it elicits allergic airway inflammation mainly via activation of ILC2, causing eosinophil infiltration, increased mucus production and AHR [112].

Regarding the interpretation of the results of studies on the protective effects of dietary fiber, it is important to establish if fiber supplementation started prior to allergen sensitization, studying allergic asthma prevention, or after sensitization but prior to allergen challenge, focusing on symptom control.

### 7.1. Preventive Effects of Dietary Fibers

Two studies with substantial impact in the research field were performed by Trompette et al., investigating effects on allergic asthma development of low-fiber, 30% pectin, and 30% cellulose diets [78] and by Thorburn et al., investigating a diet lacking fiber, and a diet with 72.7% fiber, mainly RS [117]. Using murine acute allergic HDM-mediated asthma models, it was reported that mice suffering from AAD showed, amongst other effects, lower eosinophilic airway inflammation, reduced IgE levels, and lower Th2-related inflammatory mediators compared to mice that received a control diet (Table 1), as a result of the pectin [78] or RS-fiber [117] diets, but not the cellulose diet [78]. Furthermore, airway obstruction and lung function improved, as indicated by reduced goblet cell hyperplasia, reduced mucus production, and improved AHR (Table 2). Both research groups showed that diets containing less fiber than the control chow aggravated AAD outcomes. The outcomes can possibly be explained by alterations in the microbiota composition by the fiber diets, such as relative increases of Bacteroidaceae [78,117] and Bifidobacteriaceae [78]. Additionally, increased SCFA levels were found in both the faeces and serum [78,117]. Pectin (~30%) has also been demonstrated to induce protective effects against airway inflammation, including decreased pulmonary ILC2 levels, in an IL-33-induced allergic asthma model [112]. In this last study, increased SCFA levels in the colon and lung and alterations in the faecal microbiome composition, such as increased presence of Proteobacteria and Firmicutes were shown, which are possibly involved in the mechanism behind the immunological alterations.

In these studies, diets with relatively high doses of fiber were investigated. Notably, other research groups showed similar protective effects using only low doses of other types of fermentable fiber. Verheijden et al. performed multiple studies using an HDM-induced acute allergic asthma model investigating several fibers, either with or without probiotics, including GOS (1% or 2.5% *v*/*w*) [126,127,128]) and fibers in combination with *B. breve* (*Bb*) M16V, including short-chain (sc)GOS/long-chain (lc)FOS/*Bb*(1% *w*/*w*) and scFOS/lcFOS/*Bb* (1% *w*/*w*) [129]. GOS was equally effective as the corticosteroid budesonide in abrogating mucosal mast cell activation, allergic airway eosinophilia and lymphocyte influx and activation [128]. GOS lowered pulmonary HDM-induced IL-33 levels [126] and Treg depletion studies indicated that functional Tregs contributed to the protective effect of GOS [130]. The scFOS/lcFOS/*Bb* diet, and low doses of other fibers, including low dose pectin, used in studies by other research groups, resulted in, amongst other effects, decreased eosinophilic inflammation, allergen-specific IgE, goblet cell hyperplasia, and AHR [129,131,132,133,134]. Intervention with low dose pectin also altered the microbiome composition in the faeces, characterized by increased *Lactobacillus*,* Bifidobacterium*, and *E. coli*, and decreased *Enterococcus* spp. proportions [131]. The results of these studies are the first indications that supplementation of only low doses of specific fibers might be sufficient to protect against allergic asthma development which should be considered in clinical trials. Nevertheless, studies that are more comparable are needed to draw definite conclusions. In both the high- and low-dose fiber studies mentioned above, positive outcomes on allergic asthma development were thought to be related to the microbiota-modulating effects of fiber treatments and SCFA production by the intestinal bacteria, albeit that direct evidence was only found in the studies of Trompette et al. and Thorburn et al. [78,117].

Although not shown in Table 1; Table 2, many of the aforementioned studies also showed trends of protective effects on numbers of neutrophils [127,129,131,133], macrophages [127,129], and lymphocytes [127,128,131], decreased DC activation [127,128,129], decreased levels of IL-5 [78,129,133], IL-13 [78,128,129], IL-33 [127,128,129], IgE [78,117,134], and/or improved AHR [129,134], which did not reach significance.

Interestingly, fiber intake might lead to immuno-modulatory prenatal epigenetic imprinting. An intervention with a scGOS/lcFOS diet prior to and during gestation lead to a lower acute allergic skin response, improved AHR, and declined immune infiltration in the BALF of the offspring of the dams, after the offspring were sensitized to OVA [117]. Similar results were obtained in another intervention study, in which mice were supplemented with high amylose starch or acetate (in drinking water) during gestation. Although both interventions in this study prevented AAD in the offspring, the protective effects of acetate were most robust [117].

**Table 1:** Overview of immune effects of fermentable fibers in preclinical asthma studies.

Cells and Mediators	Specific Factor of Interest Increased in AAD	Effect Dietary Fiber Compared to Control Diet	Fiber Type and Dose	Asthma Model	Reference
**Sensitization phase**					
				
				
Dendritic cells	Activation (surface expression)	↓	- 30% pectin (in PK diet 3202)	HDM	[78]
Antibody response	Total IgE	↓	- 30% pectin (in PK diet 3202)	HDM	[78]
	Allergen specific IgE	↓	**- 50/200/400 mg/kg/day AO (in water)**	OVA	[132]
			- 0.4%/kg/day pectin (i.g.) (KF chow)	OVA	[131]
	Allergen specific IgG1	↓	- 2.5% FOS	HDM	[133]
**Effector phase**					
				
Total inflammatory cells BAL		↓	**- 50 g/kg RAF, 50 g/kg GOS**	OVA	[135]
			**- 0.2 mL *Bb*/scFOS/lcFOS/AOS in PBS (i.g)**	OVA	[136]
			- 1% *v*/*w* GOS	HDM	[127]
			- 1% *w*/*w* scFOS/lcFOS (1:1) + 2% *w*/*w Bb*	HDM	[129]
			- 1% (*w*/*w*) scGOS/lcFOS (9:1), 1% (*w*/*w*) (83% scGOS/lcFOS + 17% AOS)	OVA	[134]
			- 30% pectin (in PK diet 3202)	HDM	[78]
			**- 50/200/400 mg/kg/day AO (in water)**	OVA	[132]
			- 0.4%/kg/day pectin (i.g.) (KF chow)	OVA	[131]
			- 72.7% HAMRS diet (SF11-025)	HDM	[117]
			**- 1 mg/kg SC-MN (i.n.)**	OVA	[137]
			- 1% and 2.5% GOS	HDM	[128]
Degranulating cells	Mast cells	#↓	**- 0.2 mL *Bb*/scFOS/lcFOS/AOS in PBS (i.g.)**	OVA	[136]
			- 2.5% GOS	HDM	[128]
	Eosinophils	#↓	**- 50 g/kg RAF, 50 g/kg GOS**	OVA	[135]
			- 1% *v*/*w* GOS	HDM	[127]
			- 1% *w*/*w* scFOS/lcFOS (1:1) + 2% *w*/*w Bb*	HDM	[129]
			- 30% pectin (in PK diet 3202)	HDM	[78]
			**- 50/200/400 mg/kg/day AO (in water)**	OVA	[132]
			- 0.4%/kg/day pectin (i.g.) (KF chow)	OVA	[131]
			- 72.7% HAMRS diet (SF11-025)	HDM	[117]
			- 1% and 2.5% GOS	HDM	[128]
			- 30% pectin	IL-33	[112]
			- 2.5% FOS	HDM	[133]
		%↓	**- 0.2 mL *Bb*/scFOS/lcFOS/AOS in PBS (i.g)**	OVA	[136]
			- 30% pectin	IL-33	[112]
	Macrophages	#↓	- 50/200/400 mg/kg/day AO (in water)	OVA	[132]
			- 72.7% HAMRS diet (SF11-025)	HDM	[117]
	Neutrophils	%↑	- 30% pectin	IL-33	[112]
T cells and related cells	All lymphocytes	#↓	**- 50 g/kg RAF, 50 g/kg GOS**	OVA	[135]
			- 1% *w*/*w* scFOS/lcFOS (1:1) + 2% *w*/*w Bb*	HDM	[129]
			- 72.7% HAMRS diet (SF11-025)	HDM	[117]
	Overall Th	#↓	**- 200/400 mg/kg/day AO (in water)**	OVA	[132]
	Th2	#↓	**- 400 mg/kg/day AO (in water)**	OVA	[132]
			- 30% pectin	IL-33	[112]
	ILC2	#↓	- 30% pectin	IL-33	[112]
	Th1	#↓	- 400 mg/kg/day AO (in water)	OVA	[132]
	Treg *	#↑	**- 0.2 mL *Bb*/scFOS/lcFOS/AOS in PBS (i.g.)**	OVA	[136]
Cytokines	IL-33	↓	- 1% *v*/*w* GOS	HDM	[126]
	IL-4	↓	- 30% pectin (in PK diet 3202)	HDM	[78]
			**- 50/200/400 mg/kg/day AO (in water)**	OVA	[132]
			- 0.4%/kg/day pectin (i.g.) (KF chow)	OVA	[131]
			- 72.7% HAMRS diet (SF11-025)	HDM	[117]
			- 30% pectin	IL-33	[112]
			**- 16.5 mg/kg BW LM-COS**	OVA	[138]
	IL-5	↓	**- 50 g/kg RAF**	OVA	[135]
			**- 50/200/400 mg/kg/day AO (in water)**	OVA	[132]
			- 72.7% HAMRS diet (SF11-025)	HDM	[117]
			**- 16.5 mg/kg BW LM-COS**	OVA	[138]
	IL-13	↓	- 1% *v*/*w* GOS	HDM	[127]
			**- 50/200/400 mg/kg/day AO (in water)**	OVA	[132]
			- 72.7% HAMRS diet (SF11-025)	HDM	[117]
			- 30% pectin	IL-33	[112]
			**- 16.5 mg/kg BW LM-COS**	OVA	[138]
	IFN-y	↑	- 0.4%/kg/day pectin (i.g.) (KF chow)	OVA	[131]
			- 30% pectin	IL-33	[112]
		↓	**- 50/200/400 mg/kg/day AO (in water)**	OVA	[132]
			- 72.7% HAMRS diet (SF11-025)	HDM	[117]
	IL-17	↑	- 30% pectin	IL-33	[112]
		↓	- 30% pectin (in PK diet 3202)	HDM	[78]
	IL-10	↑	**- 0.2 mL *Bb*/scFOS/lcFOS/AOS in PBS (i.g.)**	OVA	[136]
			- 0.4%/kg/day pectin (i.g.) (KF chow)	OVA	[131]
		↓	- 72.7% HAMRS diet (SF11-025)	HDM	[117]
	TNF-α	↓	**- 0.2 mL *Bb*/scFOS/lcFOS/AOS in PBS (i.g.)**	OVA	[136]
			**- 50/200/400 mg/kg/day AO (in water)**	OVA	[132]
			**- 16.5 mg/kg BW LM-COS**	OVA	[138]
	IL-1β	↓	**- 0.2 mL *Bb*/scFOS/lcFOS/AOS in PBS (i.g.)**	OVA	[136]
			**- 400 mg/kg/day AO (in water)**	OVA	[132]

Studies were included when performed in rodents. Intervention was performed with an orally administered fermentable fiber and when features related to allergic asthma were measured. All fibers were added to an AIN-93 diet, unless indicated otherwise. Outcomes from ex vivo experiments are not included. Only significant results are depicted. ↓ indicates a decrease of a specific factor by increased fiber intake. ↑ indicates an increase of a specific factor by increased fiber intake. An arrow preceded by a # or % indicates either the number of cells or percentage of cells on total cells was investigated. Fibers mentioned in bold were administered after sensitization to the allergen (symptom control). Fibers administered before sensitization to the allergen are not in bold (preventive). For each study it is indicated whether a HDM, OVA, or IL-33 asthma model was used. * This factor is decreased in AAD, instead of increased, as mentioned in title. HDM, house dust mite; OVA, ovalbumin; IL, interleukin; AAD, allergic airway disease; PK, Provimi Kliba chow; IgE, immunoglobulin E; AO, alginate oligosaccharide; KF chow, KEAOXIELE FEED chow; i.g., intragastric; IgG1, immunoglobulin G1; FOS, fructo-oligosaccharide; BAL, bronchoalveolar lavage; RAF, raffinose; GOS, galacto-oligosaccharide; *Bb*, Bifidobacterium breve; scFOS, short-chain FOS; lcFOS, long-chain FOS; AOS, pectin-derived acidic oligosaccharides; PBS, phosphate buffered saline; HAMRS, high amylose maize resistant starch; SC-MN, Saccharomyces cerevisiae derived mannan; Th, T-helper cell; ILC2, type 2 innate lymphoid cell; Treg, regulatory T-cell; LM-COS, low-molecular weight chitosan oligosaccharides; IFN-γ, interferon γ; TNF-α, tumor necrosis factor α.

**Table 2:** Overview of physiological airway effects of fermentable fibers in preclinical asthma studies.

Physiological Airway Effects	Specific Factor of Interest Increased in AAD	Effect of Dietary Fiber Compared to Control Diet	Fiber Type and Dose	Asthma Model	Reference
Airway remodeling	Epithelial denudation	↓	**- 1 mg/kg SC-MN (i.n.)**	OVA	[137]
	Surface area infiltrated with inflammatory cells	↓	**- 400 mg/kg/day AO (in water)**	OVA	[132]
			- 0.4%/kg/day pectin (i.g.) (KF chow)	OVA	[131]
			- 72.7% HAMRS diet (SF11-025)	HDM	[117]
			- 1% and 2.5% GOS	HDM	[128]
			- 2.5% FOS	HDM	[133]
	Airway smooth muscle mass	↓	**- 45 mg/kg SC-MN (i.n.)**	OVA	[137]
	Goblet cell hyperplasia or metaplasia	↓	- 30% pectin (in PK diet 3202)	HDM	[78]
			**- 400 mg/kg/day AO (in water)**	OVA	[132]
			- 0.4%/kg/day pectin (i.g.) (KF chow)	OVA	[131]
			- 72.7% HAMRS diet (SF11-025)	HDM	[117]
			- 2.5% FOS	HDM	[133]
	Mucus production	↓	- 30% pectin (in PK diet 3202)	HDM	[78]
			**- 400 mg/kg/day AO (in water)**	OVA	[132]
			**- 1 mg/kg SC-MN (i.n.)**	OVA	[137]
Airway functioning	Airway hyperresponsiveness	↓	- 1% *v*/*w* GOS	HDM	[127]
			- 30% pectin (in PK diet 3202)	HDM	[78]
			- 72.7% HAMRS diet (SF11-025)	HDM	[117]
			**- 1 mg/kg SC-MN (i.n.)**	OVA	[137]
			- 30% pectin	IL-33	[112]

Studies were included when performed in rodents. Intervention was performed with an orally administered fermentable fiber and when features related to allergic asthma were measured. All fibers were added to an AIN-93 diet, unless indicated otherwise. Outcomes from ex vivo experiments are not included. Only significant results are depicted. ↓ indicates a decrease of a specific factor by increased fiber intake. ↑ indicates an increase of a specific factor by increased fiber intake. Fibers mentioned in bold were administered after sensitization to the allergen (symptom control). Fibers administered before sensitization to the allergen are not in bold (preventive). For each study it is indicated whether a HDM, OVA, or IL-33 asthma model was used. HDM, house dust mite; OVA, ovalbumin; IL, interleukin; AAD, allergic airway disease; SC-MN, *Saccharomyces cerevisiae* derived mannan; AO, alginate oligosaccharide; KF chow, KEAOXIELE FEED chow; i.g., intragastric; HAMRS, high amylose maize resistant starch; GOS, galacto-oligosaccharide; FOS, fructo-oligosaccharide; PK, Provimi Kliba chow.

### 7.2. Symptom Control by Dietary Fibers

Several types of fibers affected allergic asthma symptom control in mouse or rat models. These include alginate oligosaccharides (AO) [132], the α-D-galactosidic linkages-containing raffinose (RAF), and GOS [135], scFOS/lcFOS/*Bb*/pectin-derived acidic-oligosaccharides (AOS) [136], and low molecular weight chitosan oligosaccharides (LM-COS) [138]. These studies were performed using OVA-induced asthma models, and all studies reported similar protective outcomes resulting from fiber supplementation. Outcomes included lower OVA-specific IgE [132], decreased numbers of total inflammatory cells [132,135,136] and eosinophils [132,135,136] in the BALF, reductions in concentrations of several inflammatory cytokines [132,135,136,138], and lower goblet cell hyperplasia and mucus production [132] (Table 1 and Table 2). Additionally, mannan derived from *Saccharomyces cerevisiae* and scFOS/lcFOS/*Bb*/AOS could counteract tissue remodelling, including airway smooth muscle (ASM) proliferation (Table 2) [136,137]. These results are the first sign that fibers could also protect against development of chronic manifestations of allergic asthma.

Multiple mechanisms of action that may explain the effects of dietary fibers have been proposed. SCFAs are likely to be involved in most of these anti-inflammatory effects via the pathways described above. Examples of other (related) possible mechanisms are modulation of innate pattern recognition receptors (PRR) [136] and suppression of MAPK signaling pathways [138]. Others have speculated that the α-D-galactosidic linkages in RAF and GOS might regulate natural killer T cells (NKT), as the resembling glycolipid α-galactosylceramide was reported to be a NKT cell ligand [135]. Whether this regulatory effect on the NKT cell would contribute to limit allergic asthma development remains to be elucidated.

## 8. Human Studies on the Effects of Dietary Fibers on Asthma

### 8.1. Epidemiological Evidence

An association between dietary fiber intake and risk for developing asthma (both allergic and non-allergic) has been found both in children [139] and in adults [140,141,142,143].

From the limited number of studies in children, two cross-sectional studies have reported inconsistent results. While Wood et al. showed no association between fiber intake and self-reported wheeze in the age group 12–18 years [144], the much larger study conducted with children between 2–12 years indicated that lower fiber intake was associated with higher odds for having asthma (Q1 vs. Q4, ever asthma OR 1.31; 95% CI 0.88–1.96, *p*-trend 0.034 and current asthma OR 1.38; 95% CI 0.87–2.20, *p*-trend 0.027) [139].

In adults with severe asthma, an inverse association between dietary fiber intake and airway eosinophilia as well as lung function decline has been reported [140]. Asthmatic adults consumed lower amounts of dietary fiber compared to non-asthmatic adults, albeit the difference appeared very small (OR 0.94 [0.90–0.99]). Fiber intake, however, was positively correlated with improved lung function parameters such as forced expiratory volume in 1 s (FEV1), vital capacity (VC), and expiratory volume (EV). These data indicate that dietary fiber may improve asthma control [140]. In adults (20–79 years of age) low dietary fiber intake was associated with a higher risk of asthma development (OR 1.4 [1.0–1.8]) compared to the highest fiber intake, especially in women and non-Hispanic white adults [141]. Similarly, a Korean study showed that the group with the highest fiber intake had the lowest prevalence of allergic asthma, which obtained statistical significance in males (OR 0.656 [0.48–0.91]) [142]. In another study, total dietary fiber intake was significantly inversely associated with asthma in both women (OR 0.73 [0.67–0.79]) and men (OR 0.63 [0.55–0.73]). Various sources of fiber were investigated and high dietary fiber intake from cereals, fruit, and seeds, was significantly associated with less asthma symptoms. Furthermore, a protective role for both soluble and insoluble fiber was suggested [143].

### 8.2. Clinical Evidence Early Life

Allergic asthma can develop following early life atopic and allergic disorders, such as atopic dermatitis (AD), allergic rhinitis, or cow’s milk allergy (atopic march) [145]. Therefore, interventions with dietary nondigestible oligosaccharides in infants with a history of atopy are needed to evaluate the preventive potential of these fibers. In one study, healthy term infants with a parental history of atopy had fewer episodes of upper respiratory tract infections and a reduced risk, compared to a placebo group, of developing recurrent wheeze after receiving a supplementation with 0.8 g/100 mL neutral oligosaccharides in the first 6 months of life [146,147,148]. Preventing early life respiratory infections and wheeze in infants with a personal or family presence of atopy, may reduce the risk of developing asthma [149]. Supplementing infant formula with the mixture GOS/lcFOS during the first 6 months of life showed similar results to the breastfed reference group with more faecal *Bifidobacterium* spp. and *Lactobacillus* spp. as well as reduced respiratory system allergic symptoms during the first 18 month of life compared to the group fed with infant formula without supplementation [150]. More recently a systematic review reported that infants exposed to dietary prebiotics show a reduced risk of developing asthma, but higher quality of evidence is needed [151].

A trial with AD infants receiving infant formula supplemented with GOS/lcFOS and *B. breve* M-16V or placebo control for 12 weeks revealed that synbiotic-supplemented formula reduced the severity of AD in patients with IgE-associated AD [152]. Intriguingly, this short period of intervention in the allergic infants reduced the prevalence of frequent wheezing, as well as the use of asthma medication, one year after the intervention [153]. In addition, compared to the placebo control, the synbiotic supplementation significantly modulated the intestinal microbiota by increasing the percentages of beneficial *Bifidobacterium* spp., while reducing unfavorable *Clostridium liuseburense*/*Clostridium histolyticum* and *Eubacterium rectale*/*Clostridium coccoides* [152,153]. Several trials in infants with suspected non-IgE mediated cow’s milk allergy [154] or with a confirmed IgE-mediated cow’s milk allergy [155] have been initiated. Here, a synbiotic blend combining scFOS/lcFOS and *B. breve* M-16V was added to infant formula which resulted in an increase in faecal *Bifidobacterium* spp. and a decrease in the *Eubacterium rectale/Clostridium coccoides* (ER/CC) group, suggesting an overall composition closer to the profile of healthy breastfed infants compared to the formula without the synbiotic blend [154,155,156]. In the light of the gut-lung-axis hypothesis and pre-clinical study outcomes, it can be hypothesized that supporting the timely colonization and diversity of the microbiome in early life could contribute to asthma prevention. Ongoing follow-up will evaluate the effects of scFOS/lcFOS and *B. breve* M-16V on the risk of developing different types of allergic diseases [157]. Future studies should provide evidence on the potential of specific dietary fibers alone or in combination with probiotics to improve immune-maturation and prevent the onset of asthma.

### 8.3. Clinical Evidence Adults

Intervention studies investigating fiber for asthma treatment have generally been performed in adults. In these studies, fiber was supplemented to investigate its effects on gut microbiome and asthma control. For this type of intervention, asthmatic subjects normally must stop or reduce their asthma medication at defined timepoints that might slightly differ between different studies. One of the first studies investigated dietary supplementation with synbiotics (8 g/day, 90% GOS/10% lcFOS with *B. breve* M-16V) in HDM-sensitized, mild asthma patients. After a 4-week intervention the peak expiratory flow (PEF) improved, while serum IL-5 reduced compared to the placebo group. In addition, ex vivo HDM re-stimulation peripheral blood mononuclear cells (PBMC) resulted in reduced Th2 type (IL-4, IL-5, and IL-13) cytokines release [158]. Prebiotic supplementation alone improved lung function using 5.5 g/day of Bimuno-galactooligosaccharide (B-GOS) in a trial with adult asthmatics. After 3 weeks of intervention, only the B-GOS-supplemented group showed improved PEF after voluntary hyperpnoea. In addition, the peak falls in FEV1 and serum TNF-α and C-reactive protein (CRP) concentrations were significantly reduced in the asthmatics following B-GOS intervention [159].

In a pilot study, patients with stable asthma were supplemented with a soluble fiber (3.5 g inulin) combined with a probiotic yoghurt (*Lactobacillus acidophilis LA5*, *L.rhamnosus GG*, and *B.lactis Bb12*) [121]. After 4 h, reduced airway inflammation was observed compared to baseline, while lung function improved compared to the control group of asthma patients. In addition, GPR41 and GPR43 gene expression in sputum samples was upregulated, implying that SCFA-mediated receptor activation may underly this protective effect in asthmatic patients [121]. This was further investigated in a trial where subjects with stable asthma received either placebo, or inulin (12 g/day) alone or in combination with a probiotic strain (same as pilot study) for 7 days [160]. Inulin alone improved the asthma control questionnaire (ACQ) score, reduced the percentage of sputum eosinophils and increased the numbers of SCFA-producing faecal bacteria. Even though the plasma SCFA levels did not change, these correlated moderately positive with the FEV1. The strongest improvement was observed in asthmatics with poorly controlled asthma and eosinophilic airway inflammation at baseline regardless of receiving inhaled corticosteroids [160].

## 9. Discussion

The link between fiber intake and immune effects in the airway is supported by many studies. Both prevention- and treatment-orientated preclinical studies with dietary fibers showed promising protective effects against allergic airway inflammation. Few studies [78,131,132,133] investigated phenotypic and functional changes in cells involved in the sensitization phase of asthma, such as IECs, DCs, or B-cells/plasma cells. Effector cells involved in symptom elicitation and inflammatory responses were investigated in more detail in all studies.

Multiple types of fiber can protect against AAD development in mice when used in a prophylactic regime, provided prior to the first sensitization and continued during allergen challenge. These include high doses of fibers such as pectin (30%) or resistant starch (72.2%) [78,112,117], but protective effects were also obtained using lower doses of fibers, such as GOS or FOS (1% or 2.5%), and combinations with *B. breve* M-16V [126,127,128,129,133,134]. The studies using high fiber amounts included a larger number of outcome parameters in the study design, but the protective effects shown on main clinical parameters, such as eosinophilic airway inflammation and AHR, were observed both using high or low doses of fibers, albeit different fibers were used in these studies. It could therefore be interesting to consider supplementation of low doses of specific fibers in clinical trials. However, more comparable studies, using similar preclinical models and outcome parameters, are needed before final conclusions can be drawn. It is noteworthy that, except for one study [131], all prophylactic murine studies made use of an acute allergic asthma model. It might be interesting to evaluate if the protective effects of fiber supplementation prior to allergen sensitization persist in the long-term. Furthermore, several clinical trials in infants have provided evidence on the potential role of dietary fibers in the prevention of atopic and allergic responses [146,147,148,150]. These examined the prebiotic combination of GOS/lcFOS in young children and found beneficial effects on both the microbiome, namely increased levels of *Bifidobacterium* spp. and *Lactobacillus* spp., as well as on the respiratory tract of the infants.

Other studies focused more on the treatment effects of fibers in mice or humans already sensitized to an airborne allergen. From preclinical studies it appeared that different types of fibers, all in quite low doses (<5% *w*/*w*), could diminish immune infiltration in the lungs, decrease multiple pro-inflammatory cytokines in the lungs and serum, and counteract airway remodeling [132,135,136,137,138]. More importantly, in infants or adults already suffering from allergic asthma or related atopic diseases different fiber types and combinations with probiotics can reduce (airway) inflammation and/or improve long function [121,152,153,158,159,160]. For inulin treatment the underlying effect mechanism possibly involved was, as in the pre-clinical studies, GPR41 and GPR43 signaling [121].

Two preclinical studies examined the effect of a combination treatment of both fibers and probiotics, such as *B. breve* [129,136]. Combinations of scGOS/lcFOS and scFOS/lcFOS with *B. breve* improved immune functioning, with scFOS/lcFOS/*Bb* showing the strongest effects [129]. Sagar et al. also found positive effects of scFOS/lcFOS with *B. breve*, although here AOS was added to the diet [136].

Based on the information discussed in this review it can be concluded that many different types of fiber have positive effects on AAD management and that SCFA potentially play an important role in the mode of action. The molecular events involved in the SCFA effects have been at least partly unravelled, being GPCR binding and HDAC inhibition, and several studies have showed the protective potential of SCFA supplementation (acetate, propionate, and butyrate in particular) on AAD-related immunological features [15,37,63,64,76,79,80,81,82,84,85,86,87,88,89,90,91,92,93,94,95,96,97,98,99,100,101,103,104,105,144,145,146,147]. As mentioned above, bacteria vary in the type and amount of enzymes they produce, and will therefore each metabolize different fibers/carbohydrates and produce different ratios of metabolites [65]. Although some preclinical studies in murine asthma models did investigate the effect of certain fibers on microbiome composition in association with asthma prevention, most studies did not investigate this. However, the prebiotic fibers that were used have already been shown clinically to modify the microbiome and to promote the growth and activity of lactobacilli and bifidobacteria [150,152,153,154,155,156]. Furthermore, concentrations of SCFAs in the faeces/cecum were not investigated in most studies, and only one study reported modulation of SCFA levels in the lung following fiber treatment [112]. Therefore, mechanistical evidence, that directly links fiber fermentation, microbiome alterations, and SCFA modulation to the protective effect of fiber on AAD disease outcomes, is still insufficient. Hence, future studies need to consider effects on the microbiome composition in the gut, as well as in the lung, and microbiome activity by measuring SCFA concentrations in the gut and lung.

To explain the effects of fibers on allergic asthma disease outcomes, mechanisms other than SCFA should also be considered. Remarkably, reduced eosinophilic infiltration in the lungs of rats, after GOS or RAF supplementation, was maintained after cecectomy and antibiotic treatment, indicating that fermentation of fibers by bacteria was not required per se to exert their immunomodulatory effects. Interestingly, the α-D-galactosidic linkages in RAF and GOS might regulate NKT cells, while FOS and xylo-oligosaccharide (XOS), that do not contain these linkages, did not exert these effects [135]. Other microbiota-independent effects might involve TLR binding and thereby the activation of downstream pathways including NF-κB, MAPK, and extracellular signal-related kinase pathways, which consequently leads to the secretion of both pro- and anti-inflammatory cytokines [161]. Another example might be that β2-1 fructans, including short chain polymers, such as FOS and long chain polymers such as inulin, could influence immune function by increasing Th1 cells in the Peyer’s Patches and DCs and Tregs frequency in the mesenteric lymph nodes of mice, without affecting the gut microbiome [162].

Furthermore, non-fermentable fibers, such as cellulose, affect the gut and microbiome [78,112,131] and even AAD development [131]. Multiple possible explanations for the effect of cellulose have been provided. These include upregulation of zinc availability in the colon by cellulose, thereby enhancing crypt proliferation, but also stimulation of goblet cell maturation by cellulose, and hence enhanced mucus secretion and increasing numbers of the mucin-degrading and ‘good health-related’ bacteria *Akkermansia muciniphila*. Lastly, cellulose might induce higher levels of niacin (vitamin B3), which is, as previously mentioned, a ligand for GPR109a just like butyrate [163]. Enhanced crypt proliferation and increased mucus secretion in the gut support a non-leaky gut. Therefore, these effects could also positively contribute to preventing asthma, as increased intestinal permeability has been linked to asthma in both children [164] and adults [165].

Altogether, dietary fibers can play a protective role in both the onset as well as symptom control of allergic asthma. Besides the possible immunostimulatory effects by the dietary fiber metabolites, SCFAs, that diminish asthma onset and severity, dietary fibers and non-fermentable fibers can potentially also directly stimulate immune functions. In the future, more clinical studies are needed to provide support to the evidence found on the protective effects of dietary fibers on allergic asthma development in early life. These studies should report on diverse immune-related parameters, but also on gut-lung microbiome composition and SCFA profiles, to obtain improved insights into the mechanisms behind the immunomodulating effects. Possible differences in the effect size of different types of fiber should receive more attention to allow for clarification of which specific dietary fiber may be the most beneficial for asthma protection, while identifying the lowest possible effective dose. Additionally, the potential of other concepts, such as synbiotic or fiber supplementation as adjunct therapies to corticosteroids (e.g., budesonide) in allergic asthma patients, deserves further investigation.

## References

[1] Barcik W., Boutin R.C., Sokolowska M., Finlay B.B. (2020). The Role of Lung and Gut Microbiota in the Pathology of Asthma. Immunity.

[2] Global Initiative for Asthma (2021). Global Strategy for Asthma Management and Prevention (2021 Update).

[3] Papi A., Brightling C., Pedersen S.E., Reddel H.K. (2018). Asthma. Lancet.

[4] Holgate S.T., Wenzel S., Postma D.S., Weiss S.T., Renz H., Sly P.D. (2015). Asthma. Nat. Rev. Dis. Primers.

[5] Mukherjee A.B., Zhang Z. (2011). Allergic asthma: Influence of genetic and environmental factors. J. Biol. Chem..

[6] Wenzel S.E. (2012). Asthma phenotypes: The evolution from clinical to molecular approaches. Nat. Med..

[7] Global Initiative for Asthma (2019). Global Strategy for Asthma Management and Prevention.

[8] Zimmermann P., Messina N., Mohn W.W., Finlay B.B., Curtis N. (2019). Association between the intestinal microbiota and allergic sensitization, eczema, and asthma: A systematic review. J. Allergy Clin. Immunol..

[9] Strachan D.P. (2000). Family size, infection and atopy: The first decade of the “hygiene hypothesis”. Thorax.

[10] Romagnani S. (1992). Human TH1 and TH2 subsets: Regulation of differentiation and role in protection and immunopathology. Int. Arch. Allergy Immunol..

[11] Bjorksten B. (2012). Diverse microbial exposure—Consequences for vaccine development. Vaccine.

[12] Haahtela T., Holgate S., Pawankar R., Akdis C.A., Benjaponpitak S., Caraballo L., Demain J., Portnoy J., von Hertzen L. (2013). The biodiversity hypothesis and allergic disease: World allergy organization position statement. World Allergy Organ. J..

[13] Spergel J.M., Paller A.S. (2003). Atopic dermatitis and the atopic march. J. Allergy Clin. Immunol..

[14] Akar-Ghibril N., Casale T., Custovic A., Phipatanakul W. (2020). Allergic Endotypes and Phenotypes of Asthma. J. Allergy Clin. Immunol. Pract..

[15] Gensollen T., Iyer S.S., Kasper D.L., Blumberg R.S. (2016). How colonization by microbiota in early life shapes the immune system. Science.

[16] Sozanska B., Sikorska-Szaflik H. (2021). Diet Modifications in Primary Prevention of Asthma. Where Do We Stand?. Nutrients.

[17] Arpaia N., Campbell C., Fan X., Dikiy S., van der Veeken J., deRoos P., Liu H., Cross J.R., Pfeffer K., Coffer P.J. (2013). Metabolites produced by commensal bacteria promote peripheral regulatory T-cell generation. Nature.

[18] Williams A.M., Probert C.S., Stepankova R., Tlaskalova-Hogenova H., Phillips A., Bland P.W. (2006). Effects of microflora on the neonatal development of gut mucosal T cells and myeloid cells in the mouse. Immunology.

[19] El Aidy S., Hooiveld G., Tremaroli V., Backhed F., Kleerebezem M. (2013). The gut microbiota and mucosal homeostasis: Colonized at birth or at adulthood, does it matter?. Gut Microbes.

[20] Arrieta M.C., Stiemsma L.T., Dimitriu P.A., Thorson L., Russell S., Yurist-Doutsch S., Kuzeljevic B., Gold M.J., Britton H.M., Lefebvre D.L. (2015). Early infancy microbial and metabolic alterations affect risk of childhood asthma. Sci. Transl. Med..

[21] Bisgaard H., Li N., Bonnelykke K., Chawes B.L., Skov T., Paludan-Muller G., Stokholm J., Smith B., Krogfelt K.A. (2011). Reduced diversity of the intestinal microbiota during infancy is associated with increased risk of allergic disease at school age. J. Allergy Clin. Immunol..

[22] Fujimura K.E., Sitarik A.R., Havstad S., Lin D.L., Levan S., Fadrosh D., Panzer A.R., LaMere B., Rackaityte E., Lukacs N.W. (2016). Neonatal gut microbiota associates with childhood multisensitized atopy and T cell differentiation. Nat. Med..

[23] Prescott S.L. (2013). Early-life environmental determinants of allergic diseases and the wider pandemic of inflammatory noncommunicable diseases. J. Allergy Clin. Immunol..

[24] Covar R.A., Strunk R., Zeiger R.S., Wilson L.A., Liu A.H., Weiss S., Tonascia J., Spahn J.D., Szefler S.J., Childhood Asthma Management Program Research Group (2010). Predictors of remitting, periodic, and persistent childhood asthma. J. Allergy Clin. Immunol..

[25] Walker W.A., Iyengar R.S. (2015). Breast milk, microbiota, and intestinal immune homeostasis. Pediatr. Res..

[26] Wan H., Winton H.L., Soeller C., Tovey E.R., Gruenert D.C., Thompson P.J., Stewart G.A., Taylor G.W., Garrod D.R., Cannell M.B. (1999). Der p 1 facilitates transepithelial allergen delivery by disruption of tight junctions. J. Clin. Investig..

[27] Hammad H., Lambrecht B.N. (2021). The basic immunology of asthma. Cell.

[28] Pichavant M., Charbonnier A.S., Taront S., Brichet A., Wallaert B., Pestel J., Tonnel A.B., Gosset P. (2005). Asthmatic bronchial epithelium activated by the proteolytic allergen Der p 1 increases selective dendritic cell recruitment. J. Allergy Clin. Immunol..

[29] Lambrecht B.N., Hammad H. (2015). The immunology of asthma. Nat. Immunol..

[30] Ballesteros-Tato A., Randall T.D., Lund F.E., Spolski R., Leonard W.J., Leon B. (2016). T Follicular Helper Cell Plasticity Shapes Pathogenic T Helper 2 Cell-Mediated Immunity to Inhaled House Dust Mite. Immunity.

[31] Coquet J.M., Schuijs M.J., Smyth M.J., Deswarte K., Beyaert R., Braun H., Boon L., Karlsson Hedestam G.B., Nutt S.L., Hammad H. (2015). Interleukin-21-Producing CD4(+) T Cells Promote Type 2 Immunity to House Dust Mites. Immunity.

[32] Peebles R.S., Aronica M.A. (2019). Proinflammatory Pathways in the Pathogenesis of Asthma. Clin. Chest Med..

[33] Tindemans I., van Schoonhoven A., KleinJan A., de Bruijn M.J., Lukkes M., van Nimwegen M., van den Branden A., Bergen I.M., Corneth O.B., van I.W.F. (2020). Notch signaling licenses allergic airway inflammation by promoting Th2 cell lymph node egress. J. Clin. Investig..

[34] Eckl-Dorna J., Villazala-Merino S., Campion N.J., Byazrova M., Filatov A., Kudlay D., Karsonova A., Riabova K., Khaitov M., Karaulov A. (2019). Tracing IgE-Producing Cells in Allergic Patients. Cells.

[35] Tindemans I., Serafini N., Di Santo J.P., Hendriks R.W. (2014). GATA-3 function in innate and adaptive immunity. Immunity.

[36] Klose C.S., Artis D. (2016). Innate lymphoid cells as regulators of immunity, inflammation and tissue homeostasis. Nat. Immunol..

[37] Van der Ploeg E.K., Carreras Mascaro A., Huylebroeck D., Hendriks R.W., Stadhouders R. (2020). Group 2 Innate Lymphoid Cells in Human Respiratory Disorders. J. Innate. Immun..

[38] Li B.W., de Bruijn M.J., Tindemans I., Lukkes M., KleinJan A., Hoogsteden H.C., Hendriks R.W. (2016). T cells are necessary for ILC2 activation in house dust mite-induced allergic airway inflammation in mice. Eur. J. Immunol..

[39] Guo L., Huang Y., Chen X., Hu-Li J., Urban J.F., Paul W.E. (2015). Innate immunological function of TH2 cells in vivo. Nat. Immunol..

[40] Nobs S.P., Kayhan M., Kopf M. (2019). GM-CSF intrinsically controls eosinophil accumulation in the setting of allergic airway inflammation. J. Allergy Clin. Immunol..

[41] Ramakrishnan R.K., Al Heialy S., Hamid Q. (2019). Role of IL-17 in asthma pathogenesis and its implications for the clinic. Expert Rev. Respir. Med..

[42] Koch S., Sopel N., Finotto S. (2017). Th9 and other IL-9-producing cells in allergic asthma. Semin. Immunopathol..

[43] Akbari O., Stock P., DeKruyff R.H., Umetsu D.T. (2003). Role of regulatory T cells in allergy and asthma. Curr. Opin. Immunol..

[44] Martin-Orozco E., Norte-Munoz M., Martinez-Garcia J. (2017). Regulatory T Cells in Allergy and Asthma. Front. Pediatr..

[45] Dang A.T., Marsland B.J. (2019). Microbes, metabolites, and the gut-lung axis. Mucosal. Immunol..

[46] Martinez-Guryn K., Leone V., Chang E.B. (2019). Regional Diversity of the Gastrointestinal Microbiome. Cell Host Microbe.

[47] Zoetendal E.G., Raes J., van den Bogert B., Arumugam M., Booijink C.C., Troost F.J., Bork P., Wels M., de Vos W.M., Kleerebezem M. (2012). The human small intestinal microbiota is driven by rapid uptake and conversion of simple carbohydrates. ISME J..

[48] Rodriguez J.M., Murphy K., Stanton C., Ross R.P., Kober O.I., Juge N., Avershina E., Rudi K., Narbad A., Jenmalm M.C. (2015). The composition of the gut microbiota throughout life, with an emphasis on early life. Microb. Ecol. Health Dis..

[49] Cukrowska B., Bierla J.B., Zakrzewska M., Klukowski M., Maciorkowska E. (2020). The Relationship between the Infant Gut Microbiota and Allergy. The Role of Bifidobacterium breve and Prebiotic Oligosaccharides in the Activation of Anti-Allergic Mechanisms in Early Life. Nutrients.

[50] Solis G., de Los Reyes-Gavilan C.G., Fernandez N., Margolles A., Gueimonde M. (2010). Establishment and development of lactic acid bacteria and bifidobacteria microbiota in breast-milk and the infant gut. Anaerobe.

[51] Morris A., Beck J.M., Schloss P.D., Campbell T.B., Crothers K., Curtis J.L., Flores S.C., Fontenot A.P., Ghedin E., Huang L. (2013). Comparison of the respiratory microbiome in healthy nonsmokers and smokers. Am. J. Respir. Crit. Care Med..

[52] Bassis C.M., Erb-Downward J.R., Dickson R.P., Freeman C.M., Schmidt T.M., Young V.B., Beck J.M., Curtis J.L., Huffnagle G.B., Ravel J. (2015). Analysis of the Upper Respiratory Tract Microbiotas as the Source of the Lung and Gastric Microbiotas in Healthy Individuals. MBio.

[53] Pattaroni C., Watzenboeck M.L., Schneidegger S., Kieser S., Wong N.C., Bernasconi E., Pernot J., Mercier L., Knapp S., Nicod L.P. (2018). Early-Life Formation of the Microbial and Immunological Environment of the Human Airways. Cell Host Microbe.

[54] Marsland B.J., Gollwitzer E.S. (2014). Host-microorganism interactions in lung diseases. Nat. Rev. Immunol..

[55] Samuelson D.R., Welsh D.A., Shellito J.E. (2015). Regulation of lung immunity and host defense by the intestinal microbiota. Front. Microbiol..

[56] Schuijt T.J., Lankelma J.M., Scicluna B.P., de Sousa e Melo F., Roelofs J.J., de Boer J.D., Hoogendijk A.J., de Beer R., de Vos A., Belzer C. (2016). The gut microbiota plays a protective role in the host defence against pneumococcal pneumonia. Gut.

[57] Brandt E.B., Scribner T.A., Akei H.S., Rothenberg M.E. (2006). Experimental gastrointestinal allergy enhances pulmonary responses to specific and unrelated allergens. J. Allergy Clin. Immunol..

[58] Navarro S., Cossalter G., Chiavaroli C., Kanda A., Fleury S., Lazzari A., Cazareth J., Sparwasser T., Dombrowicz D., Glaichenhaus N. (2011). The oral administration of bacterial extracts prevents asthma via the recruitment of regulatory T cells to the airways. Mucosal. Immunol..

[59] Pivniouk V., Gimenes-Junior J.A., Ezeh P., Michael A., Pivniouk O., Hahn S., VanLinden S.R., Malone S.P., Abidov A., Anderson D. (2021). Airway administration of OM-85, a bacterial lysate, blocks experimental asthma by targeting dendritic cells and the epithelium/IL-33/ILC2 axis. J. Allergy Clin. Immunol..

[60] Akdis C.A., Akdis M. (2011). Mechanisms of allergen-specific immunotherapy. J. Allergy Clin. Immunol..

[61] Ruane D., Brane L., Reis B.S., Cheong C., Poles J., Do Y., Zhu H., Velinzon K., Choi J.H., Studt N. (2013). Lung dendritic cells induce migration of protective T cells to the gastrointestinal tract. J. Exp. Med..

[62] Tulic M.K., Piche T., Verhasselt V. (2016). Lung-gut cross-talk: Evidence, mechanisms and implications for the mucosal inflammatory diseases. Clin. Exp. Allergy.

[63] Budden K.F., Gellatly S.L., Wood D.L., Cooper M.A., Morrison M., Hugenholtz P., Hansbro P.M. (2017). Emerging pathogenic links between microbiota and the gut-lung axis. Nat. Rev. Microbiol..

[64] Mutlu E.A., Comba I.Y., Cho T., Engen P.A., Yazici C., Soberanes S., Hamanaka R.B., Nigdelioglu R., Meliton A.Y., Ghio A.J. (2018). Inhalational exposure to particulate matter air pollution alters the composition of the gut microbiome. Environ. Pollut..

[65] Holscher H.D. (2017). Dietary fiber and prebiotics and the gastrointestinal microbiota. Gut Microbes.

[66] Stephen A.M., Champ M.M., Cloran S.J., Fleith M., van Lieshout L., Mejborn H., Burley V.J. (2017). Dietary fibre in Europe: Current state of knowledge on definitions, sources, recommendations, intakes and relationships to health. Nutr. Res. Rev..

[67] Desai M.S., Seekatz A.M., Koropatkin N.M., Kamada N., Hickey C.A., Wolter M., Pudlo N.A., Kitamoto S., Terrapon N., Muller A. (2016). A Dietary Fiber-Deprived Gut Microbiota Degrades the Colonic Mucus Barrier and Enhances Pathogen Susceptibility. Cell.

[68] Bridgman S.L., Azad M.B., Field C.J., Haqq A.M., Becker A.B., Mandhane P.J., Subbarao P., Turvey S.E., Sears M.R., Scott J.A. (2017). Fecal Short-Chain Fatty Acid Variations by Breastfeeding Status in Infants at 4 Months: Differences in Relative versus Absolute Concentrations. Front. Nutr..

[69] Cummings J.H., Pomare E.W., Branch W.J., Naylor C.P., Macfarlane G.T. (1987). Short chain fatty acids in human large intestine, portal, hepatic and venous blood. Gut.

[70] Sivaprakasam S., Bhutia Y.D., Yang S., Ganapathy V. (2017). Short-Chain Fatty Acid Transporters: Role in Colonic Homeostasis. Compr. Physiol..

[71] Koh A., De Vadder F., Kovatcheva-Datchary P., Backhed F. (2016). From Dietary Fiber to Host Physiology: Short-Chain Fatty Acids as Key Bacterial Metabolites. Cell.

[72] Frost G., Sleeth M.L., Sahuri-Arisoylu M., Lizarbe B., Cerdan S., Brody L., Anastasovska J., Ghourab S., Hankir M., Zhang S. (2014). The short-chain fatty acid acetate reduces appetite via a central homeostatic mechanism. Nat. Commun..

[73] Segal L.N., Clemente J.C., Li Y., Ruan C., Cao J., Danckers M., Morris A., Tapyrik S., Wu B.G., Diaz P. (2017). Anaerobic Bacterial Fermentation Products Increase Tuberculosis Risk in Antiretroviral-Drug-Treated HIV Patients. Cell Host Microbe.

[74] Mirkovic B., Murray M.A., Lavelle G.M., Molloy K., Azim A.A., Gunaratnam C., Healy F., Slattery D., McNally P., Hatch J. (2015). The Role of Short-Chain Fatty Acids, Produced by Anaerobic Bacteria, in the Cystic Fibrosis Airway. Am. J. Respir. Crit. Care Med..

[75] Ghorbani P., Santhakumar P., Hu Q., Djiadeu P., Wolever T.M., Palaniyar N., Grasemann H. (2015). Short-chain fatty acids affect cystic fibrosis airway inflammation and bacterial growth. Eur. Respir. J..

[76] Blad C.C., Tang C., Offermanns S. (2012). G protein-coupled receptors for energy metabolites as new therapeutic targets. Nat. Rev. Drug Discov..

[77] Theiler A., Barnthaler T., Platzer W., Richtig G., Peinhaupt M., Rittchen S., Kargl J., Ulven T., Marsh L.M., Marsche G. (2019). Butyrate ameliorates allergic airway inflammation by limiting eosinophil trafficking and survival. J. Allergy Clin. Immunol..

[78] Trompette A., Gollwitzer E.S., Yadava K., Sichelstiel A.K., Sprenger N., Ngom-Bru C., Blanchard C., Junt T., Nicod L.P., Harris N.L. (2014). Gut microbiota metabolism of dietary fiber influences allergic airway disease and hematopoiesis. Nat. Med..

[79] Steinmeyer S., Lee K., Jayaraman A., Alaniz R.C. (2015). Microbiota metabolite regulation of host immune homeostasis: A mechanistic missing link. Curr. Allergy Asthma Rep..

[80] Sealy L., Chalkley R. (1978). The effect of sodium butyrate on histone modification. Cell.

[81] Adcock I.M., Tsaprouni L., Bhavsar P., Ito K. (2007). Epigenetic regulation of airway inflammation. Curr. Opin. Immunol..

[82] Stadhouders R., Filion G.J., Graf T. (2019). Transcription factors and 3D genome conformation in cell-fate decisions. Nature.

[83] Cavalli G., Heard E. (2019). Advances in epigenetics link genetics to the environment and disease. Nature.

[84] Creyghton M.P., Cheng A.W., Welstead G.G., Kooistra T., Carey B.W., Steine E.J., Hanna J., Lodato M.A., Frampton G.M., Sharp P.A. (2010). Histone H3K27ac separates active from poised enhancers and predicts developmental state. Proc. Natl. Acad. Sci. USA.

[85] Meyers D.A., Bleecker E.R., Holloway J.W., Holgate S.T. (2014). Asthma genetics and personalised medicine. Lancet Respir. Med..

[86] Moffatt M.F., Gut I.G., Demenais F., Strachan D.P., Bouzigon E., Heath S., von Mutius E., Farrall M., Lathrop M., Cookson W. (2010). A large-scale, consortium-based genomewide association study of asthma. N. Engl. J. Med..

[87] Liang L., Willis-Owen S.A.G., Laprise C., Wong K.C.C., Davies G.A., Hudson T.J., Binia A., Hopkin J.M., Yang I.V., Grundberg E. (2015). An epigenome-wide association study of total serum immunoglobulin E concentration. Nature.

[88] Yang I.V., Pedersen B.S., Liu A., O’Connor G.T., Teach S.J., Kattan M., Misiak R.T., Gruchalla R., Steinbach S.F., Szefler S.J. (2015). DNA methylation and childhood asthma in the inner city. J. Allergy Clin. Immunol..

[89] Seumois G., Chavez L., Gerasimova A., Lienhard M., Omran N., Kalinke L., Vedanayagam M., Ganesan A.P., Chawla A., Djukanovic R. (2014). Epigenomic analysis of primary human T cells reveals enhancers associated with TH2 memory cell differentiation and asthma susceptibility. Nat. Immunol..

[90] Sheikhpour M., Maleki M., Ebrahimi Vargoorani M., Amiri V. (2021). A review of epigenetic changes in asthma: Methylation and acetylation. Clin. Epigenet..

[91] Stadhouders R., Li B.W.S., de Bruijn M.J.W., Gomez A., Rao T.N., Fehling H.J., van I.W.F.J., Lim A.I., Di Santo J.P., Graf T. (2018). Epigenome analysis links gene regulatory elements in group 2 innate lymphocytes to asthma susceptibility. J. Allergy Clin. Immunol..

[92] Akdis C.A. (2021). Does the epithelial barrier hypothesis explain the increase in allergy, autoimmunity and other chronic conditions?. Nat. Rev. Immunol..

[93] Martin-Gallausiaux C., Marinelli L., Blottiere H.M., Larraufie P., Lapaque N. (2021). SCFA: Mechanisms and functional importance in the gut. Proc. Nutr. Soc..

[94] Richards L.B., Li M., Folkerts G., Henricks P.A.J., Garssen J., van Esch B. (2020). Butyrate and Propionate Restore the Cytokine and House Dust Mite Compromised Barrier Function of Human Bronchial Airway Epithelial Cells. Int. J. Mol. Sci..

[95] Wawrzyniak P., Wawrzyniak M., Wanke K., Sokolowska M., Bendelja K., Ruckert B., Globinska A., Jakiela B., Kast J.I., Idzko M. (2017). Regulation of bronchial epithelial barrier integrity by type 2 cytokines and histone deacetylases in asthmatic patients. J. Allergy Clin. Immunol..

[96] Steelant B., Wawrzyniak P., Martens K., Jonckheere A.C., Pugin B., Schrijvers R., Bullens D.M., Vanoirbeek J.A., Krawczyk K., Dreher A. (2019). Blocking histone deacetylase activity as a novel target for epithelial barrier defects in patients with allergic rhinitis. J. Allergy Clin. Immunol..

[97] Millard A.L., Mertes P.M., Ittelet D., Villard F., Jeannesson P., Bernard J. (2002). Butyrate affects differentiation, maturation and function of human monocyte-derived dendritic cells and macrophages. Clin. Exp. Immunol..

[98] Cait A., Hughes M.R., Antignano F., Cait J., Dimitriu P.A., Maas K.R., Reynolds L.A., Hacker L., Mohr J., Finlay B.B. (2018). Microbiome-driven allergic lung inflammation is ameliorated by short-chain fatty acids. Mucosal. Immunol..

[99] Berndt B.E., Zhang M., Owyang S.Y., Cole T.S., Wang T.W., Luther J., Veniaminova N.A., Merchant J.L., Chen C.C., Huffnagle G.B. (2012). Butyrate increases IL-23 production by stimulated dendritic cells. Am. J. Physiol. Gastrointest. Liver Physiol..

[100] Luu M., Pautz S., Kohl V., Singh R., Romero R., Lucas S., Hofmann J., Raifer H., Vachharajani N., Carrascosa L.C. (2019). The short-chain fatty acid pentanoate suppresses autoimmunity by modulating the metabolic-epigenetic crosstalk in lymphocytes. Nat. Commun..

[101] Mendez-Enriquez E., Hallgren J. (2019). Mast Cells and Their Progenitors in Allergic Asthma. Front. Immunol..

[102] Folkerts J., Redegeld F., Folkerts G., Blokhuis B., van den Berg M.P.M., de Bruijn M.J.W., van I.W.F.J., Junt T., Tam S.Y., Galli S.J. (2020). Butyrate inhibits human mast cell activation via epigenetic regulation of FcepsilonRI-mediated signaling. Allergy.

[103] Rada-Iglesias A., Enroth S., Ameur A., Koch C.M., Clelland G.K., Respuela-Alonso P., Wilcox S., Dovey O.M., Ellis P.D., Langford C.F. (2007). Butyrate mediates decrease of histone acetylation centered on transcription start sites and down-regulation of associated genes. Genome Res..

[104] Krajewski D., Kaczenski E., Rovatti J., Polukort S., Thompson C., Dollard C., Ser-Dolansky J., Schneider S.S., Kinney S.R.M., Mathias C.B. (2018). Epigenetic Regulation via Altered Histone Acetylation Results in Suppression of Mast Cell Function and Mast Cell-Mediated Food Allergic Responses. Front. Immunol..

[105] Richon V.M., Sandhoff T.W., Rifkind R.A., Marks P.A. (2000). Histone deacetylase inhibitor selectively induces p21WAF1 expression and gene-associated histone acetylation. Proc. Natl. Acad. Sci. USA.

[106] Thio C.L., Chi P.Y., Lai A.C., Chang Y.J. (2018). Regulation of type 2 innate lymphoid cell-dependent airway hyperreactivity by butyrate. J. Allergy Clin. Immunol..

[107] Park J., Kim M., Kang S.G., Jannasch A.H., Cooper B., Patterson J., Kim C.H. (2015). Short-chain fatty acids induce both effector and regulatory T cells by suppression of histone deacetylases and regulation of the mTOR-S6K pathway. Mucosal. Immunol..

[108] Vieira R.S., Castoldi A., Basso P.J., Hiyane M.I., Camara N.O.S., Almeida R.R. (2019). Butyrate Attenuates Lung Inflammation by Negatively Modulating Th9 Cells. Front. Immunol..

[109] Wen T., Aronow B.J., Rochman Y., Rochman M., Kc K., Dexheimer P.J., Putnam P., Mukkada V., Foote H., Rehn K. (2019). Single-cell RNA sequencing identifies inflammatory tissue T cells in eosinophilic esophagitis. J. Clin. Investig..

[110] Kespohl M., Vachharajani N., Luu M., Harb H., Pautz S., Wolff S., Sillner N., Walker A., Schmitt-Kopplin P., Boettger T. (2017). The Microbial Metabolite Butyrate Induces Expression of Th1-Associated Factors in CD4(+) T Cells. Front. Immunol..

[111] Kabata H., Moro K., Koyasu S. (2018). The group 2 innate lymphoid cell (ILC2) regulatory network and its underlying mechanisms. Immunol. Rev..

[112] Lewis G., Wang B., Shafiei Jahani P., Hurrell B.P., Banie H., Aleman Muench G.R., Maazi H., Helou D.G., Howard E., Galle-Treger L. (2019). Dietary Fiber-Induced Microbial Short Chain Fatty Acids Suppress ILC2-Dependent Airway Inflammation. Front. Immunol..

[113] Toki S., Goleniewska K., Reiss S., Zhou W., Newcomb D.C., Bloodworth M.H., Stier M.T., Boyd K.L., Polosukhin V.V., Subramaniam S. (2016). The histone deacetylase inhibitor trichostatin A suppresses murine innate allergic inflammation by blocking group 2 innate lymphoid cell (ILC2) activation. Thorax.

[114] Sepahi A., Liu Q., Friesen L., Kim C.H. (2021). Dietary fiber metabolites regulate innate lymphoid cell responses. Mucosal. Immunol..

[115] Kasal D.N., Liang Z., Hollinger M.K., O’Leary C.Y., Lisicka W., Sperling A.I., Bendelac A. (2021). A Gata3 enhancer necessary for ILC2 development and function. Proc. Natl. Acad. Sci. USA.

[116] Furusawa Y., Obata Y., Fukuda S., Endo T.A., Nakato G., Takahashi D., Nakanishi Y., Uetake C., Kato K., Kato T. (2013). Commensal microbe-derived butyrate induces the differentiation of colonic regulatory T cells. Nature.

[117] Thorburn A.N., McKenzie C.I., Shen S., Stanley D., Macia L., Mason L.J., Roberts L.K., Wong C.H., Shim R., Robert R. (2015). Evidence that asthma is a developmental origin disease influenced by maternal diet and bacterial metabolites. Nat. Commun..

[118] Smith P.M., Howitt M.R., Panikov N., Michaud M., Gallini C.A., Bohlooly Y.M., Glickman J.N., Garrett W.S. (2013). The microbial metabolites, short-chain fatty acids, regulate colonic Treg cell homeostasis. Science.

[119] Faith J.J., Ahern P.P., Ridaura V.K., Cheng J., Gordon J.I. (2014). Identifying gut microbe-host phenotype relationships using combinatorial communities in gnotobiotic mice. Sci. Transl. Med..

[120] Vinolo M.A., Hatanaka E., Lambertucci R.H., Newsholme P., Curi R. (2009). Effects of short chain fatty acids on effector mechanisms of neutrophils. Cell Biochem. Funct..

[121] Halnes I., Baines K.J., Berthon B.S., MacDonald-Wicks L.K., Gibson P.G., Wood L.G. (2017). Soluble Fibre Meal Challenge Reduces Airway Inflammation and Expression of GPR43 and GPR41 in Asthma. Nutrients.

[122] Chang P.V., Hao L., Offermanns S., Medzhitov R. (2014). The microbial metabolite butyrate regulates intestinal macrophage function via histone deacetylase inhibition. Proc. Natl. Acad. Sci. USA.

[123] Zhu L., Qin S., Zhai S., Gao Y., Li L. (2017). Inulin with different degrees of polymerization modulates composition of intestinal microbiota in mice. FEMS Microbiol. Lett..

[124] Nials A.T., Uddin S. (2008). Mouse models of allergic asthma: Acute and chronic allergen challenge. Dis. Model. Mech..

[125] Conrad M.L., Yildirim A.O., Sonar S.S., Kilic A., Sudowe S., Lunow M., Teich R., Renz H., Garn H. (2009). Comparison of adjuvant and adjuvant-free murine experimental asthma models. Clin. Exp. Allergy.

[126] Verheijden K.A., Akbari P., Willemsen L.E., Kraneveld A.D., Folkerts G., Garssen J., Fink-Gremmels J., Braber S. (2015). Inflammation-induced expression of the alarmin interleukin 33 can be suppressed by galacto-oligosaccharides. Int. Arch. Allergy Immunol..

[127] Verheijden K.A., Willemsen L.E., Braber S., Leusink-Muis T., Delsing D.J., Garssen J., Kraneveld A.D., Folkerts G. (2015). Dietary galacto-oligosaccharides prevent airway eosinophilia and hyperresponsiveness in a murine house dust mite-induced asthma model. Respir. Res..

[128] Verheijden K.A.T., Braber S., Leusink-Muis T., Jeurink P.V., Thijssen S., Kraneveld A.D., Garssen J., Folkerts G., Willemsen L.E.M. (2018). The Combination Therapy of Dietary Galacto-Oligosaccharides With Budesonide Reduces Pulmonary Th2 Driving Mediators and Mast Cell Degranulation in a Murine Model of House Dust Mite Induced Asthma. Front. Immunol..

[129] Verheijden K.A., Willemsen L.E., Braber S., Leusink-Muis T., Jeurink P.V., Garssen J., Kraneveld A.D., Folkerts G. (2016). The development of allergic inflammation in a murine house dust mite asthma model is suppressed by synbiotic mixtures of non-digestible oligosaccharides and Bifidobacterium breve M-16V. Eur. J. Nutr..

[130] Verheijden K.A., Braber S., Leusink-Muis T., Thijssen S., Boon L., Kraneveld A.D., Garssen J., Folkerts G., Willemsen L.E. (2015). Regulatory T Cell Depletion Abolishes the Protective Effect of Dietary Galacto-Oligosaccharides on Eosinophilic Airway Inflammation in House Dust Mite-Induced Asthma in Mice. J. Nutr..

[131] Zhang Z., Shi L., Pang W., Liu W., Li J., Wang H., Shi G. (2016). Dietary Fiber Intake Regulates Intestinal Microflora and Inhibits Ovalbumin-Induced Allergic Airway Inflammation in a Mouse Model. PLoS ONE.

[132] Bang M.A., Seo J.H., Seo J.W., Jo G.H., Jung S.K., Yu R., Park D.H., Park S.J. (2015). Bacillus subtilis KCTC 11782BP-produced alginate oligosaccharide effectively suppresses asthma via T-helper cell type 2-related cytokines. PLoS ONE.

[133] Yasuda A., Inoue K.I., Sanbongi C., Yanagisawa R., Ichinose T., Yoshikawa T., Takano H. (2010). Dietary supplementation with fructooligosaccharides attenuates airway inflammation related to house dust mite allergen in mice. Int. J. Immunopathol. Pharmacol..

[134] Vos A.P., van Esch B.C., Stahl B., M’Rabet L., Folkerts G., Nijkamp F.P., Garssen J. (2007). Dietary supplementation with specific oligosaccharide mixtures decreases parameters of allergic asthma in mice. Int. Immunopharmacol..

[135] Sonoyama K., Watanabe H., Watanabe J., Yamaguchi N., Yamashita A., Hashimoto H., Kishino E., Fujita K., Okada M., Mori S. (2005). Allergic airway eosinophilia is suppressed in ovalbumin-sensitized Brown Norway rats fed raffinose and alpha-linked galactooligosaccharide. J. Nutr..

[136] Sagar S., Vos A.P., Morgan M.E., Garssen J., Georgiou N.A., Boon L., Kraneveld A.D., Folkerts G. (2014). The combination of Bifidobacterium breve with non-digestible oligosaccharides suppresses airway inflammation in a murine model for chronic asthma. Biochim. Biophys. Acta.

[137] Lew D.B., Michael C.F., Overbeck T., Robinson W.S., Rohman E.L., Lehman J.M., Patel J.K., Eiseman B., LeMessurier K.S., Samarasinghe A.E. (2017). Beneficial Effects of Prebiotic Saccharomyces cerevisiae Mannan on Allergic Asthma Mouse Models. J. Immunol. Res..

[138] Chung M.J., Park J.K., Park Y.I. (2012). Anti-inflammatory effects of low-molecular weight chitosan oligosaccharides in IgE-antigen complex-stimulated RBL-2H3 cells and asthma model mice. Int. Immunopharmacol..

[139] Vaccaro J.A., Niego J., Huffman F.G. (2016). Dietary factors, body weight, and screen time in U.S. children with and without asthma. Child. Health Care.

[140] Berthon B.S., Macdonald-Wicks L.K., Gibson P.G., Wood L.G. (2013). Investigation of the association between dietary intake, disease severity and airway inflammation in asthma. Respirology.

[141] Saeed M.A., Gribben K.C., Alam M., Lyden E.R., Hanson C.K., LeVan T.D. (2020). Association of Dietary Fiber on Asthma, Respiratory Symptoms, and Inflammation in the Adult National Health and Nutrition Examination Survey Population. Ann. Am. Thorac. Soc..

[142] Lee H., Lee K., Son S., Kim Y.C., Kwak J.W., Kim H.G., Lee S.H., Kim T.H. (2021). Association of Allergic Diseases and Related Conditions with Dietary Fiber Intake in Korean Adults. Int. J. Environ. Res. Public Health.

[143] Andrianasolo R.M., Hercberg S., Kesse-Guyot E., Druesne-Pecollo N., Touvier M., Galan P., Varraso R. (2019). Association between dietary fibre intake and asthma (symptoms and control): Results from the French national e-cohort NutriNet-Sante. Br. J. Nutr..

[144] Wood L.G., Lagleva M., Shah S., Berthon B.S., Galbraith S., Henry R., Kepreotes H., Gibson P.G. (2015). Dietary changes in migrant adolescents with increasing length of stay in Australia and associated risk of wheeze—A retrospective, cross sectional study. BMC Pediatr..

[145] Gabryszewski S.J., Hill D.A. (2021). One march, many paths: Insights into allergic march trajectories. Ann. Allergy Asthma Immunol..

[146] Arslanoglu S., Moro G.E., Boehm G., Wienz F., Stahl B., Bertino E. (2012). Early neutral prebiotic oligosaccharide supplementation reduces the incidence of some allergic manifestations in the first 5 years of life. J. Biol. Regul. Homeost. Agents.

[147] Arslanoglu S., Moro G.E., Schmitt J., Tandoi L., Rizzardi S., Boehm G. (2008). Early dietary intervention with a mixture of prebiotic oligosaccharides reduces the incidence of allergic manifestations and infections during the first two years of life. J. Nutr..

[148] Moro G., Arslanoglu S., Stahl B., Jelinek J., Wahn U., Boehm G. (2006). A mixture of prebiotic oligosaccharides reduces the incidence of atopic dermatitis during the first six months of age. Arch. Dis. Child..

[149] Beigelman A., Bacharier L.B. (2016). Early-life respiratory infections and asthma development: Role in disease pathogenesis and potential targets for disease prevention. Curr. Opin. Allergy Clin. Immunol..

[150] Ivakhnenko O.S., Nyankovskyy S.L. (2013). Effect of the specific infant formula mixture of oligosaccharides on local immunity and development of allergic and infectious disease in young children: Randomized study. Pediatr. Polska.

[151] Cuello-Garcia C., Fiocchi A., Pawankar R., Yepes-Nunez J.J., Morgano G.P., Zhang Y., Agarwal A., Gandhi S., Terracciano L., Schunemann H.J. (2017). Prebiotics for the prevention of allergies: A systematic review and meta-analysis of randomized controlled trials. Clin. Exp. Allergy.

[152] Van der Aa L.B., Heymans H.S., van Aalderen W.M., Sillevis Smitt J.H., Knol J., Ben Amor K., Goossens D.A., Sprikkelman A.B., Synbad Study G. (2010). Effect of a new synbiotic mixture on atopic dermatitis in infants: A randomized-controlled trial. Clin. Exp. Allergy.

[153] Van der Aa L.B., van Aalderen W.M., Heymans H.S., Henk Sillevis Smitt J., Nauta A.J., Knippels L.M., Ben Amor K., Sprikkelman A.B., Synbad Study G. (2011). Synbiotics prevent asthma-like symptoms in infants with atopic dermatitis. Allergy.

[154] Candy D.C.A., Van Ampting M.T.J., Oude Nijhuis M.M., Wopereis H., Butt A.M., Peroni D.G., Vandenplas Y., Fox A.T., Shah N., West C.E. (2018). A synbiotic-containing amino-acid-based formula improves gut microbiota in non-IgE-mediated allergic infants. Pediatr. Res..

[155] Chatchatee P., Nowak-Wegrzyn A., Lange L., Benjaponpitak S., Chong K.W., Sangsupawanich P., van Ampting M.T.J., Oude Nijhuis M.M., Harthoorn L.F., Langford J.E. (2021). Tolerance development in cow’s milk-allergic infants receiving amino acid-based formula: A randomized controlled trial. J. Allergy Clin. Immunol..

[156] Wopereis H., van Ampting M.T.J., Cetinyurek-Yavuz A., Slump R., Candy D.C.A., Butt A.M., Peroni D.G., Vandenplas Y., Fox A.T., Shah N. (2019). A specific synbiotic-containing amino acid-based formula restores gut microbiota in non-IgE mediated cow’s milk allergic infants: A randomized controlled trial. Clin. Transl. Allergy.

[157] NederlandsTrialRegister Identifier NTR3567, a Prospective Double Blind Randomised Controlled Study to Evaluate the Immunological Benefits and Clinical Effects of an Elimination Diet Using an Amino Acid Formula (AAF) with an Added Pre-probiotic Blend in Infants with Cow’s Milk Allergy (CMA). https://www.trialregister.nl/trial/3567.

[158] van de Pol M.A., Lutter R., Smids B.S., Weersink E.J., van der Zee J.S. (2011). Synbiotics reduce allergen-induced T-helper 2 response and improve peak expiratory flow in allergic asthmatics. Allergy.

[159] Williams N.C., Johnson M.A., Shaw D.E., Spendlove I., Vulevic J., Sharpe G.R., Hunter K.A. (2016). A prebiotic galactooligosaccharide mixture reduces severity of hyperpnoea-induced bronchoconstriction and markers of airway inflammation. Br. J. Nutr..

[160] McLoughlin R., Berthon B.S., Rogers G.B., Baines K.J., Leong L.E.X., Gibson P.G., Williams E.J., Wood L.G. (2019). Soluble fibre supplementation with and without a probiotic in adults with asthma: A 7-day randomised, double blind, three way cross-over trial. EBioMedicine.

[161] Pujari R., Banerjee G. (2021). Impact of prebiotics on immune response: From the bench to the clinic. Immunol. Cell Biol..

[162] Fransen F., Sahasrabudhe N.M., Elderman M., Bosveld M., El Aidy S., Hugenholtz F., Borghuis T., Kousemaker B., Winkel S., van der Gaast-de Jongh C. (2017). beta2-->1-Fructans Modulate the Immune System In Vivo in a Microbiota-Dependent and -Independent Fashion. Front. Immunol..

[163] Kim Y., Hwang S.W., Kim S., Lee Y.S., Kim T.Y., Lee S.H., Kim S.J., Yoo H.J., Kim E.N., Kweon M.N. (2020). Dietary cellulose prevents gut inflammation by modulating lipid metabolism and gut microbiota. Gut Microbes.

[164] Hijazi Z., Molla A.M., Al-Habashi H., Muawad W.M., Molla A.M., Sharma P.N. (2004). Intestinal permeability is increased in bronchial asthma. Arch. Dis. Child..

[165] Benard A., Desreumeaux P., Huglo D., Hoorelbeke A., Tonnel A.B., Wallaert B. (1996). Increased intestinal permeability in bronchial asthma. J. Allergy Clin. Immunol..

